# Telemedical support for prehospital emergency medical service in severe emergencies: an open-label randomised non-inferiority clinical trial

**DOI:** 10.1186/s13054-023-04545-z

**Published:** 2023-06-30

**Authors:** Ana Kowark, Marc Felzen, Sebastian Ziemann, Stephanie Wied, Michael Czaplik, Stefan K. Beckers, Jörg C. Brokmann, Ralf-Dieter Hilgers, Rolf Rossaint, the TEMS-study group, J. Bartman, L. Becker, L. Bozlu, M. Coburn, G. Fazlipour, C. Fitzner, L. Grüßer, G.-A. Gunesch, P. Hess, M. Holten, E. Junge, Dennis Juppen, S. Kaffanke, T. Koch, P. Kranke, J. Liebens, M. Müller, Stephan Ortmanns, Martin Reugels, Ute Roschanski, Jane Schroeder, Pia Stadler, Carla Tutlies, Julia Van Waesberghe

**Affiliations:** 1 Department of Anaesthesiology, Medical Faculty, University Hospital RWTH Aachen, Aachen, Germany; 2 Department of Anaesthesiology and Intensive Care Medicine, University Hospital Bonn, Bonn, Germany; 3 Department of Medical Statistics, Medical Faculty, University Hospital RWTH Aachen, Aachen, Germany; 4 Emergency Department, Medical Faculty, University Hospital RWTH Aachen, Aachen, Germany; 5 Marien-Hospital, Aachen, Germany; 6 Luisen-Hospital, Aachen, Germany; 7 University Hospital Bonn, Bonn, Germany; 8 Franziskus-Hospital, Aachen, Germany; 9 University Hospital RWTH Aachen, Aachen, Germany; 10 Rhein-Maas-Klinikum, Würselen, Germany; 11 University Hospital Dresden, Dresden, Germany; 12 University Hospital Würzburg, Würzburg, Germany

**Keywords:** Adverse events in pre-hospital emergencies, Emergency medical service, Remote emergency physician, Tele-emergency medical service, Telemedicine

## Abstract

**Background:**

A tele-emergency medical service with a remote emergency physician for severe prehospital emergencies may overcome the increasing number of emergency calls and shortage of emergency medical service providers. We analysed whether routine use of a tele-emergency medical service is non-inferior to a conventional physician-based one in the occurrence of intervention-related adverse events.

**Methods:**

This open-label, randomised, controlled, parallel-group, non-inferiority trial included all routine severe emergency patients aged ≥ 18 years within the ground-based ambulance service of Aachen, Germany. Patients were randomised in a 1:1 allocation ratio to receive either tele-emergency medical service (*n* = 1764) or conventional physician-based emergency medical service (*n* = 1767). The primary outcome was the occurrence of intervention-related adverse events with suspected causality to the group assignment. The trial was registered with ClinicalTrials.gov (NCT02617875) on 30 November 2015 and is reported in accordance with the CONSORT statement for non-inferiority trials.

**Results:**

Among 3531 randomised patients, 3220 were included in the primary analysis (mean age, 61.3 years; 53.8% female); 1676 were randomised to the conventional physician-based emergency medical service (control) group and 1544 to the tele-emergency medical service group. A physician was not deemed necessary in 108 of 1676 cases (6.4%) and 893 of 1544 cases (57.8%) in the control and tele-emergency medical service groups, respectively. The primary endpoint occurred only once in the tele-emergency medical service group. The Newcombe hybrid score method confirmed the non-inferiority of the tele-emergency medical service, as the non-inferiority margin of − 0.015 was not covered by the 97.5% confidence interval of − 0.0046 to 0.0025.

**Conclusions:**

Among severe emergency cases, tele-emergency medical service was non-inferior to conventional physician-based emergency medical service in terms of the occurrence of adverse events.

**Supplementary Information:**

The online version contains supplementary material available at 10.1186/s13054-023-04545-z.

## Introduction

Telemedicine is a continuously evolving and useful healthcare service that improves patient outcomes by increasing equity, quality, cost-effectiveness, and particularly timely access to care [1]. In prehospital emergency setting, transfer of patient data to the hospital shortens the time span to effective in-hospital treatment for patients with stroke and myocardial infarction [2–4]. Moreover, many countries have observed an increasing number of emergency calls due to the growing aging population, a shortage of emergency medical service (EMS) providers, and—in countries with a conventional physician-based EMS—a prolongation of the physician response time to severe and life-threatening emergencies. In many European and Asian countries [5] with a conventional physician-based EMS, both the medical dispatch protocol of the dispatching centre and its specification when to dispatch an EMS physician in addition to the ambulance staffed with paramedics are still heterogeneous. In Germany [6], there is an obligation to dispatch an EMS physician for life-threatening emergencies as well as for severe emergencies with potentially need for drug administration. However, there is often a discrepancy between the described situation during the emergency call to the dispatcher and the real one, resulting in dispatching an EMS physician without any indication. This problem could be reduced by the possibility of teleconsultation for the ambulance in several cases [1, 7].

After developing a tele-EMS for all different kinds of emergencies and performing various implementation, feasibility, non-inferiority, and safety trials between 2007 and 2013, a 24/7 health insurance-reimbursed tele-EMS was added complementary to the conventional physician-based EMS in 2014 for the routine care of prehospital emergencies in Aachen, Germany [8–13].

To date, there has been no randomised controlled trial (RCT) allocating emergency calls either to a tele-EMS or a conventional physician-based EMS [14]. Therefore, the Telemedical support for prehospital emergency medical service (TEMS) trial aimed to evaluate whether primary dispatching of the tele-EMS compared to the conventional physician-based EMS is non-inferior in the occurrence of system-induced patient adverse events (AEs). The non-inferiority design was chosen based on the expectation that non-inferiority of the tele-EMS for severe emergencies would provide more efficiency in the treatment of a wider range of emergency cases and consequently save the EMS-physician resource. Secondary, we hypothesised that the tele-EMS physician would be superior to a conventional EMS physician on scene in terms of anamnesis, documentation, and treatment quality, despite a shorter physician engagement time [9, 11, 13, 15, 16].

## Methods

### Ethics statements

The institutional ethics committee of the University Hospital Aachen approved this trial on 25 November 2015 (approval number: EK 170/15). The ethics committee waived the need for informed consent for the initial randomisation and EMS-treatment, and it was only required for the follow-up procedures.

### Study design

This open-label, randomised, controlled, parallel-group, non-inferiority trial with two interim analyses and no trial adaptation was conducted within the ground-based emergency medical district of Aachen. All resulting EMS files and hospital information system-based outcome data of admitted patients were analysed within the scope of data quality assurance for outcome measures. Patients admitted to five hospitals (one university hospital and four primary care hospitals) were followed. The latest version of the published study protocol [17] and amended trial statistical analysis plan (TSAP) are presented in Additional file 1 and Additional file 2, respectively. The TSAP was finalised before database export and analysis. The trial was registered with ClinicalTrials.gov (NCT02617875) on 30 November 2015 and is reported in accordance with the CONSORT statement for non-inferiority trials [18]. Additional methods are presented in Additional file 3.

The data safety monitoring committee reviewed non-blinded safety data. The study director at the University Hospital RWTH Aachen was responsible for study conduction.

### Participants

We included all routine patients aged ≥ 18 years with severe (such as acute coronary syndrome, stroke or hypertensive emergency) emergencies. Acute life-threatening emergencies (such as polytrauma, respiratory insufficiency, apnoea or cardio circulatory arrest) [17] were excluded as for these patients an EMS physician on scene cannot be denied because of potentially required manual skills (such as intubation, chest drainage or resuscitation). Enrolment occurred independent of the final hospital destination or decision for conveyance to the emergency department.

### Teleconsultation system

Technical details are described elsewhere [19]. In brief, the tele-EMS transfers all vital, audio, and video data from the emergency scene to a teleconsultation centre equipped with an EMS physician and supported by software (TM-Documentation, Umlaut Telehealthcare GmbH, Aachen, Germany) using the latest checklists and guidelines.

All paramedics on a telemedically-equipped ambulance received a one-day training focused on indication for teleconsultation and telemedical equipment. Every conventional EMS physician is at least in the 4th year of residency in anaesthesiology and passed an examination in emergency medicine. In addition, every tele-EMS physician is a board-certified anaesthetist and completed at least 500 on-site emergency missions combined with further training in telemedicine.

Paramedics are obliged to call the tele-EMS physician 24/7, if drugs or invasive procedures are to be carried out according to an SOP, as they are not allowed to carry out them alone according to German law [13, 19, 20]. If the tele-EMS physician is not accessible, a physician on site is mandatory by law in such cases.

### Randomisation, study procedure, and interventions

During the emergency call, the dispatchers in the central dispatching centre of the fire department in Aachen typed the suspected symptoms into the dispatching software (Cobra 4, ISE GmbH, Aachen, Germany). An algorithm in the dispatching software initiated randomisation automatically if essential operational conditions were met, e.g. availability of an EMS physician in both study arms and the entered symptom matched a severe condition according to the EMS physician indication catalogue of the German federal medical association. Patients were allocated to receive either tele-EMS (tele-group) or conventional physician-based EMS (control group) using complete randomisation with a 1:1 allocation ratio. Before implementation of the tele-EMS physician for each of the patients, an EMS-physician was alerted. Since implementation, a parallel alert can only be dispensed because the paramedics can consult the tele-EMS physician by pushing a button. The dispatcher assigned the patients to their respective groups as randomised. A manual conversion of the initial assignment in terms of the tele-group was required, if in the further course of the phone call the dispatcher assumed a life-threatening emergency. Further, the initially dispatched team was always allowed to pass the mission to the other group on both sides based on the situational assessment.

### Tele-EMS group

Paramedics were initially dispatched in a ground-based ambulance alone and were authorised to decide based on their situational assessment and specifications in standard operating procedures (SOPs), whether they needed tele-EMS physician involvement for the respective patient. However, if the patient unexpectedly presented on scene with an acute life-threatening condition, meeting the exclusion criteria, they were obliged to alert an EMS-physician and inform the tele-EMS physician. Whether they have appropriately followed this SOP was not directly assessed in this study, but indirectly by the occurrence of AEs.

### Control group

Paramedics in a ground-based ambulance and the conventional EMS physician in a separate vehicle were dispatched simultaneously in a so-called rendezvous system and treated the patients on scene as in the clinical routine. If paramedics arrived first on scene, they were also authorised to cancel the involvement of a conventional EMS physician if not needed.

### Tele- and control groups

A standardised paramedic-EMS file was completed by paramedics, and if involved, also a physician-EMS file by physicians in both groups.

Blinding was not possible because of the obvious treatment procedure. A follow-up was carried out on days 30 and 90 after the intervention.

### Outcomes

The primary outcome with respect to the non-inferiority of the tele-group was the frequency of four predefined intervention-related AEs during EMS treatment with suspected causality to the group assignment, as one would expect benefit or minimising risk having an EMS physician on scene.

The predefined AEs, which were not the original reason for the emergency call, were chosen by the authors because they represent the most common AEs associated with drug therapy in the EMS and comprised the following three new immediate AEs: (i) allergic reaction (documented in the free text or indicated by the use of anti-allergic drugs after any treatment by the EMS team); (ii) blood pressure decrease (necessity of vasopressors); and (iii) respiratory insufficiency (decrease in peripheral oxygen saturation < 90%, or need for assistive, manual or controlled ventilation). Further, it comprised one new AE with a time lag of 24 h, in order to capture also delayed complications possibly resulting from not guideline-conform treatment of the EMS: (iv) cardiac arrest within 24 h of the intervention. The term “intervention-related” covered beside wrong treatments (e.g. iatrogenic allergic reaction because of inadequate survey of medical history; iatrogenic blood pressure drop, apnoea or respiratory insufficiency after wrong drug dosage or selection; or circulatory arrest after wrong drug dosage, or wrong hospital referral) also the cases where the respective EMS did not provide any treatment. An independent clinical endpoint committee (CEC) judged whether the reported AEs were suspected to have occurred because of group assignment based on blinded data (see also Additional file 3).

Secondary outcomes regarding the superiority hypotheses for the tele-group were analysed by means of the EMS files and comprised the guideline-conform medical history survey, treatment and documentation quality, AEs independent of the EMS care used, and the necessity of a physician for the EMS operation. For further details on outcome measures, see Additional file 1 and Additional file 2. The proportion of fulfilled SAMPLE acronym [21] items (consisting of the survey of S = Symptoms, A = Allergies, M = Medication, P = Past pertinent medical history, L = Last oral intake, and E = Events leading to present Illness/Injury) indicated the quality of the medical history survey, which was assessed by the proportion of documented patient vital data in accordance with the respective guidelines [22]. Treatment quality was evaluated by the frequency of adherence to six predefined clinically relevant components (correct hospital choice, electrocardiography (ECG) use, intravenous access placement, non-invasive blood pressure, oxygen saturation, and blood glucose level). The secondary outcomes evaluated by means of automatically generated reports of the dispatching software comprised the physician engagement time and further operating times, including cancellation of the EMS physician or conversions between the groups. The secondary outcomes death within 24-h, 30-days, 90-days and hospitalisation; discharge destination; intensive care unit and hospital length of stay were analysed using the EMS files, hospital database, and follow-up interviews.

### Sample size and statistical analysis

Data are presented as means and standard deviations (SDs) for continuous variables, and as frequencies and percentages for categorical variables. Because of the lack of evidence for EMS-dependent AEs, our sample size calculation was based on analysis of our own data with an assumed AE rate of 2% for the conventional physician-based EMS. We assumed a non-inferiority margin of 1.5% and a power of 80%, and we allocated an overall significance level of 5% to *K* = 3, as two interim analyses for non-inferiority were planned [23]. (Additional file 3). All AE cases were included in the interim analyses of the primary endpoint (Additional file 2).

The final analysis of all randomised cases was performed at a significance level of 2.25%. Considering an assumed dropout rate of 10% evenly distributed over the stages and groups, the calculated sample size using Addplan 6.0 [24] was 3344 patients or 1672 per group. Statistical analyses were performed using SAS (version 9.4; SAS Institute Inc.). To evaluate the non-inferiority of the tele-group regarding the rate of causal AEs, the respective 99.97% and 99.29% confidence intervals (CIs) for the rate difference between the groups were calculated (proc FREQ in SAS). For the final evaluation of the primary endpoint, the 97.75% CI of rate differences (proc FREQ in SAS) was calculated using the Newcombe hybrid score method, as only one causal AE was observed in the tele-group.

To evaluate all dichotomous secondary endpoints, we used two-sided chi-square tests (proc FREQ). The exact Fisher test was applied for values < 5, and no further evaluation was performed in the case of 0 events in both groups. All continuous endpoints were evaluated with two-sided t-tests (proc TTEST), and the bootstrap statement with 10 000 bootstrap samples was used to calculate the bootstrap t intervals for skewed data.

Except for the primary endpoint, *p*-values < 5% were considered statistically significant. Deviations from the original TSAP are described in the Additional file 3.

## Results

### Study population

**Fig. 1:**
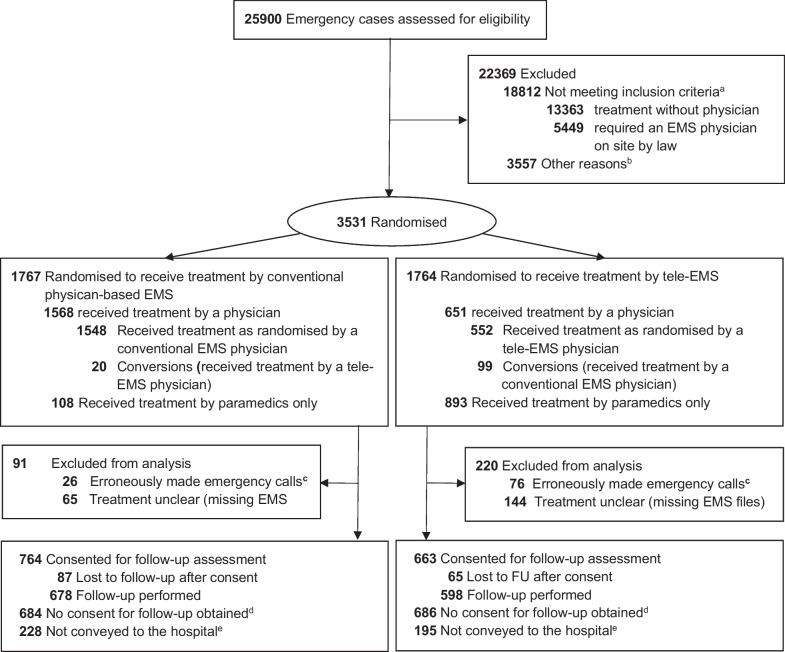
Participant flow in the TEMS trial. EMS = emergency medical service. ^a^*n* = 13,363 cases, which were treated solely by paramedics and did not require an EMS physician according to the standard dispatching criteria and *n* = 5449 cases, which obligatory required an EMS physician on scene according to the standard dispatching criteria. ^b^This refers to the cases, which met the inclusion criteria, but could not be randomised due to a lack of treatment capacity in at least one of the randomisation arms. ^c^Erroneously made emergency calls without any EMS treatment or patient at scene. ^d^Examples: Patient refused consent or a consent could not be obtained due to organisational reasons. ^e^In Germany, a written informed consent could only be obtained personally by a physician. Thus, a written informed consent for the follow-up assessments could only be sought in both groups for conveyed patients to the hospital

From 13 August 2018 to 5 September 2019, 3531 of 25,900 out-of-hospital emergency patients were enrolled and randomised into either the control group (*n* = 1767) or tele-group (*n* = 1764) (Fig. 1). A manual conversion of the assigned treatment group was performed in 119 cases by the dispatching personnel or the initially dispatched team (*n* = 99 were switched from tele-EMS to conventional physician-based EMS treatment; *n* = 20 vice versa). The paramedics cancelled the involvement of the respective EMS-physician after arrival at the emergency scene because of their own situational judgment in 108 of 1676 cases (6.4%) and 893 of 1544 cases (57.8%) in the control and tele-groups, respectively. Thus, 1568 and 651 patients received treatment by an EMS-physician in the control and tele-groups, respectively. A total of 3220 patients were analysed for the primary endpoint (Fig. 1).

Tables 1 and 2 present the data for the entire population and the cases with physician contact. The baseline characteristics were similar in both groups (Tables 1; Additional file 3: Table S1). Patients had a mean age of 61.3 years and 53.8% were female.

**Table 1:** Baseline characteristics in the TEMS trial

	Overall (*n* = 3220)	Control group (*n* = 1676)	Tele-group (*n* = 1544)	All cases with physician contact (*n* = 2219)	Control group with physician contact (*n* = 1568)	Tele-group with physician contact (*n* = 651)
Age, median (range), *y*	66 (0–103)	66 (0–103)	66 (0–100)	67 (0–103)	66 (0–103)	69 (1–98)
*Sex*, No. (%)^a^						
Female	1733 (53.8)	911 (54.4)	822 (53.2)	1206 (54.6)	851 (54.3)	355 (54.5)
Male	1478 (45.9)	759 (45.3)	719 (46.6)	1008 (45.4)	713 (45.5)	295 (45.3)
Missing values	9 (0.3)	6 (0.4)	3 (0.2)	5 (0.2)	4 (0.3)	1 (0.2)
*Initially suspected symptom complex, *No. (%)^a,b^						
Acute coronary syndrome	388 (12.1)	211 (12.6)	177 (11.5)	321 (14.5)	207 (13.2)	114 (17.5)
Stroke without unconsciousness	314 (9.8)	157 (9.4)	157 (10.2)	246 (11.1)	149 (9.5)	97 (14.9)
Slight dyspnoea	306 (9.5)	146 (8.7)	160 (10.4)	192 (8.7)	135 (8.6)	57 (8.8)
Acute unclear abdomen	261 (8.1)	150 (9.0)	111 (7.2)	178 (8.0)	144 (9.2)	34 (5.2)
Syncope	166 (5.2)	87 (5.2)	79 (5.1)	109 (4.9)	84 (5.4)	25 (3.8)
Back pain	125 (3.9)	66 (3.9)	59 (3.8)	87 (3.9)	62 (4.0)	25 (3.8)
Neurologic emergency case	121 (3.8)	69 (4.1)	52 (3.4)	84 (3.8)	66 (4.2)	18 (2.8)
Arterial Hypertension	117 (3.6)	64 (3.8)	53 (3.4)	88 (4.0)	62 (4.0)	26 (4.0)
Hypertensive emergency	117 (3.6)	57 (3.4)	60 (3.9)	79 (3.6)	55 (3.5)	24 (3.7)
Thoracic pain	116 (3.6)	66 (3.9)	50 (3.2)	83 (3.7)	64 (4.1)	19 (2.9)
NACA score first diagnosis,^a,c,e^	NA	NA	NA	*n* = 1867^d^	*n* = 1308^d^	*n* = 559^d^
Mean (SD)	NA	NA	NA	3.1 (0.7)	3.1 (0.7)	3.2 (0.8)
NACA score last diagnosis,^a,c,e^	NA	NA	NA	*n* = 1763^d^	*n* = 1217^d^	*n* = 546^d^
Mean (SD)	NA	NA	NA	3.1 (0.7)	3.0 (0.7)	3.2 (0.8)

*NA* not applicable; *NACA* National Advisory Committee for Aeronautics; *min* minutes; *SD* standard deviation

^a^Percentages may not add to 100% due to rounding

^b^Entered into the dispatching software by the staff in the dispatching centre during the interview of the caller

^c^Only applicable for the cases with physician contact, as it is not assessed in the paramedic emergency medical service files

^d^Total number is deviating from the total number indicated in the column heading due to missing data

^e^Representing the mean and SD of the NACA 2–7 scores (NACA 2 = moderate disturbance, no hospital admission necessary; NACA 3 = severe but not life-threatening; NACA 4 = potentially life-threatening; NACA 5 = acute risk of death; NACA 6 = successful cardiopulmonary resuscitation; NACA 7 = death)

**Table 2:** Clinical outcomes in the TEMS trial

	Control group (*n* = 1676)	Tele-group (*n* = 1544)	Mean difference or risk difference^a^ (95% CI)	*P* value	Control group with physician contact (*n* = 1568)	Tele-group with physician contact (*n* = 651)	Mean difference or risk difference^a^ (95% CI)	*P* value
*Primary outcome*								
Total frequency of the four predefined intervention-related^b^ AEs during the EMS treatment with causality to the group assignment, No. (%)	0 (0)	1 (0.1)^c^	− 0.0046 to 0.0025^d^		NA	NA	NA	
*Secondary outcomes*								
Quality of medical history survey^e^								
Mean (SD),%	71.4 (23.8)	72.4 (24.8)	1.0 (− 0.7 to 2.7)	0.24	71.8 (23.4)	80.9 (16.3)	9.1 (7.4–10.8)	< 0.01
S—symptoms, No. (%)	1627 (97.1)	1457 (94.4)	NA	NA	1525 (97.3)	639 (98.2)	NA	NA
A—allergy, No. (%)	1024 (61.1)	1137 (73.7)	NA	NA	960 (61.2)	581 (89.3)	NA	NA
M—Medication, No. (%)	1189 (70.9)	1204 (78.0)	NA	NA	1118 (71.3)	604 (92.8)	NA	NA
P—past medical history, No. (%)	1313 (78.3)	1178 (76.3)	NA	NA	1248 (79.6)	603 (92.6)	NA	NA
L—last oral intake, No. (%)	612 (36.5)	499 (32.3)	NA	NA	569 (36.3)	161 (24.7)	NA	NA
E—events prior incident, No. (%)	1416 (84.5)	1234 (79.9)	NA	NA	1332 (85.0)	572 (87.9)	NA	NA
Quality of medical history survey regarding the assessment of “Patient’s Risk factors”, No. (%)^f^	679 (40.5)	537 (34.8)	− 5.7 (2.4–9.1)	< 0.01	663 (42.3)	327 (50.2)	8.0 (3.4–12.5)	< 0.01
*Quality of documentation*^g^								
Mean (SD), %	74.0 (20.1)	66.4 (25.3)	− 7.6 (− 9.2 to − 6.0)	< 0.01	75.8 (18.2)	84.5 (12.2)	8.7 (7.4–10.0)	< 0.01
Treatment quality (adherence to guidelines)								
Correct hospital choice, No. (%)	1422 (84.8)	1323 (85.7)	0.8 (− 3.3 to 1.6)	0.50	1339 (85.4)	590 (90.6)	5.2 (2.4–8.1)	< 0.01
ECG used, No. (%)	1343 (80.1)	1060 (68.7)	11.5 (8.4–14.5)	< 0.01	1296 (82.7)	609 (93.6)	10.9 (8.2–13.6)	< 0.01
NIBP measured, No. (%)	1568 (93.6)	1419 (91.9)	1.7 (− 0.2 to 4.0)	0.07	1493 (95.2)	641 (98.5)	3.6 (1.8–4.7)	< 0.01
SpO_2_ measured, No. (%)	1577 (94.1)	1455 (94.2)	− 0.1 (− 1.2 to 1.5)	0.86	1487 (94.8)	635 (97.5)	2.7 (2.2–4.3)	0.01
Blood glucose level measured, No. (%)	1169 (69.8)	904 (58.6)	11.2 (7.9–14.5)	< 0.01	1138 (72.6)	502 (77.1)	4.5 (0.6–8.5)	0.03
Intravenous access, No. (%)	907 (54.1)	538 (34.8)	19.3 (15.9–22.6)	< 0.01	899 (57.3)	452 (69.4)	12.1 (7.8–16.4)	< 0.01
Duration of the physician engagement-time^h^	NA	NA	NA		*n* = 1527^i^	*n* = 494^i^		
Mean (SD), min	NA	NA	NA		28.2 (13.2)	16.5 (18.2)	− 11.7 (− 13.3 to − 9.8)	NA^j^
Termination of the EMS physician involvement, as deemed unnecessary, No. (%)	108 (6.4)	893 (57.8)	51.4 (48.7–54.1)	< 0.01	0 (0)	0 (0)	NA	NA

*AEs* adverse events; *CI* confidence interval; *ECG* electrocardiogram; *EMS* emergency medical service; *NA* not applicable; *NIBP* non-invasive blood pressure; *No.* number; *SD* standard deviation; *SpO*_*2*_ oxygen saturation as measured by pulse oxymetry; *IQR* interquartile range

^a^Mean difference for continuous variables and risk difference for categorical variables

^b^Intervention-related means that the AE occurred iatrogenic, e.g. allergic reaction because of inadequate survey of medical history; blood pressure drop, apnoea or respiratory insufficiency on scene (e.g. after wrong drug dosage or drug selection); circulatory arrest within 24 h of EMS treatment (e.g. after wrong drug dosage, drug selection, or wrong hospital referral)

^c^This adverse event was an iatrogenic cardio-circulatory arrest within 24 h of the intervention

^d^97.75% Confidence interval according to the Newcombe hybrid-score method

^e^Percentage adherence to the SAMPLE-acronym. 100% corresponds to a survey of all 6 SAMPLE items (S = Symptoms, A = Allergies, M = Medication, P = Past pertinent medical history, L = Last oral intake and E = Events leading to present Illness/Injury)

^f^Assessed by the frequency of any assessed and documented risk factor

^g^The documentation quality was assessed by the proportion of documented patient vital data in accordance with the respective guidelines (Please refer to the TSAP in Additional file 2)

^h^Physician engagement time was defined as the timespan between the alarm (assignment to the group) and the operational readiness at the end of the mission

^i^Total number is deviating from the total number indicated in the column heading due to missing data

^j^No *p*-value calculated as the bootstrap method was used

### Primary outcome

The statistical analysis using the Newcombe hybrid score method revealed that the non-inferiority margin of − 0.015 was not covered by the 97.75% CI of − 0.0046 to 0.0025. Thus, the non-inferiority hypothesis could be confirmed (Table 2), indicating that tele-EMS is non-inferior to the conventional physician-based EMS. Only one of the intervention-related AEs had suspected causality to the group assignment. This AE occurred in the tele-group (a patient with cancellation due to tele-EMS involvement and treatment by paramedics only). A hypertensive emergency was not medically treated in the prehospital setting as the guidelines would have demanded, and the patient had a cardiac arrest within 24 h of the emergency call due to intracranial bleeding.

### Secondary outcomes

The secondary outcomes are presented in Tables 2 and 3 and Additional file 3: Tables S2–S4.

**Table 3:** Follow-up data after hospital admission

	Control group (*n* = 1676)	Tele-group (*n* = 1544)	Mean difference or risk difference^a^ (95% CI)	*P* value
Hospital length of stay	*n* = 1411	*n* = 1317		
Mean (SD), days	4.3 (6.8)	4.6 (6.8)	0.3 (− 0.2 to 0.8)	NA^b^
Intensive care unit length of stay	*n* = 144	*n* = 150		
Mean (SD), days	5.6 (8.3)	5.6 (8.1)	0.1 (− 1.8 to 2.0)	NA^b^
Death within 24 h,	*n* = 993	*n* = 901		
No. (%)	8 (0.8)	10 (1.1)	0.3 (− 0.6 to 1.2)	0.50
Death until hospital discharge	*n* = 1402	*n* = 1236		
No. (%)	25 (1.8)	34 (2.7)	0.9 (− 0.3 to 2.0)	0.14
Death within 30 days	*n* = 692	*n* = 616		
No. (%)	23 (3.3)	17 (2.8)	− 0.6 (− 2.4 to 1.3)	0.55
Death within 90 days	*n* = 675	*n* = 598		
No. (%)	36 (5.3)	30 (5.0)	− 0.3 (− 2.8 to 2.1)	0.80

*CI* confidence interval; *NA* not applicable; *No.* number; *SD* standard deviation

^a^Mean difference for continuous variables and risk difference for categorical variables

^b^97.75% Confidence interval according to the Newcombe hybrid-score method

#### All cases

Only 42% of 1544 cases in the tele-group had EMS physician contact, compared to 94% of 1676 in the control group (Table 2). There were no differences in all assessed parameters, apart from a better performance in the control group than in the tele-group regarding a conventional physician-based more frequent assessment of risk factors, use of ECG monitoring, measurement of blood glucose levels, application of an intravenous access, and better documentation (Table 2). The mean time from randomisation to hospital arrival was with 37.4 (10.4) versus 39.3 (13.9) min (*p* < 0.001) significantly shorter in the control group than in the tele-group (Additional file 3: Table S4). The follow-up data after hospital admission were similar between the groups (Table 3).

#### Cases with physician contact

Comparison of only the cases with physician contact showed that compared to the control group, the tele-group had significantly better quality of the medical history survey and documentation quality, and the patients received better treatment quality (Table 2).

The physician engagement time was significantly shorter in the tele-group than in the control group (Table 2).

## Discussion

In this RCT, we revealed that prehospital emergency care using a tele-EMS is non-inferior to the conventional physician-based EMS in severe but not acute life-threatening emergencies in the occurrence of intervention-related AEs with causality to the group assignment. Moreover, patients in the tele-group could be managed by the paramedics alone in 58% of all cases. If a physician was deemed necessary and became involved, the tele-EMS physician was superior in terms of several secondary outcomes, as he/she gets the diagnostic/therapeutic guideline and disease-specific SOP displayed on screen and hence is not at risk of missing anything basic.

With a new German Emergency Paramedic law passed in 2014, competencies for paramedics without an EMS-physician on site were permitted, which were previously prohibited by law. A relevant decrease in the use of EMS-physicians with regard to severe missions is only expected in the future, since not all paramedics have been trained or feel save accordingly. Nevertheless, the consultation of a tele-EMS-physician can provide low-threshold support for the paramedics in the event of problems, since not all assignments can be mapped in the form of SOPs. With the current level of training of the paramedics of a Franco-German EMS system [25], a tele-EMS system offers advantages over a pure paramedic system even if some publications show no advantage in terms of non-life-threatening missions [26]. Notwithstanding that sufficient randomised controlled trials regarding prehospital treatment of physicians and paramedics are not yet available, some publications show advantages for the care of the rarely occurring life-threatening missions by EMS physicians [27, 28].

Although not outcome relevant, the time span between the emergency call and arrival at the hospital was shorter with patients treated by a conventional physician-based EMS than those with tele-EMS. Nevertheless, a complete “SAMPLE”-anamnesis combined with a telephone pre-registration by the tele-EMS-physician helps to speed up further treatment in the hospital [29, 30]. For cases with tele-EMS physician treatment, it is most likely that either paramedics first examined the patient at the emergency site or they had taken the patient to the ambulance before contacting the tele-EMS physician. This is further resource saving while being non-inferior to the conventional group, where the physician on scene is directly involved in the initial patient assessment using routine non-invasive monitoring and/or in the transportation of the patient to the ambulance.

To our best knowledge, this is the first large RCT to compare tele-EMS with conventional EMS for severe but not acute life-threatening cases within a municipal EMS routine. Previous studies were either non-randomised [16, 19, 20, 31] or restricted to a specific patient condition [32, 33]. This study also investigated two different prehospital EMS systems.

Our results demonstrated that even though the paramedics cancelled the tele-EMS physician involvement in 58% of the emergency cases, the emergency care in this tele-EMS was safe without significantly more AEs. Interestingly, the threshold for cancellation of physician involvement by paramedics was significantly higher in the control group, while the severity and type of cases were similar in both groups. This is because of that the conventional EMS physician arrived in 293 and 455 cases before and in parallel with the paramedics on scene, respectively. In the remaining cases, the mean time difference was only 4:51 min, which impeded the paramedics to adequately evaluate the emergency before the physician arrived. Contrary, in the tele-group the tele-EMS physician was always available by pushing a button and does not drive to scene; tele-EMS physician was only not cancelled, if there was real medical necessity. Hence, primary dispatching of tele-EMS for severe emergencies does not withhold the legally prescribed EMS-physician but might save the limited resources of a conventional EMS physician and allow focused dispatching of conventional EMS physicians to acute life-threatening emergencies [13].

A higher quality and shorter physician engagement time for the complementarily used tele-EMS physician was previously shown [13] and could also be confirmed within the TEMS trial for severe emergencies.

The TEMS trial demonstrated safe, feasible, and high-quality patient-centred use of telemedicine in prehospital emergency settings, which might be relevant and quality improving for all EMS systems [34]. Our results indicate that the AEs and outcomes in the tele- and control group were not different, suggesting that the decision to cancel tele-EMS physician involvement was generally acceptable.

## Limitations

To mitigate selection bias, complete randomisation was used to balance allocation. Nevertheless, we cannot explain the difference in number of erroneous calls between the two groups.

Detection bias could not be excluded, as knowledge of the randomisation might have affected documentation of the primary endpoint, although a blinded assessment was implemented. In addition, paramedic EMS files differed from physician files, which might have led to different documentation for patients without physician contact per se and might have introduced performance bias. In cases without physician contact, we could only evaluate the paramedic EMS-file. The higher number of missing EMS-files in the tele-group might be explained by the lower willingness among the paramedics to write a protocol than among physicians.

We acknowledge a relatively high loss to follow-up for the secondary 30- and 90-day outcome. One reason is that in Germany only a physically present physician may obtain a written informed consent. Thus, it could only be sought in both groups for conveyed patients to the hospital, albeit it was missed in several cases due to organisational reasons.

One major limitation is the discrepancy between our estimated and observed number of AEs.

Further, our secondary outcomes have to be considered carefully, as our study was not powered for any of them. Moreover, we acknowledge that our results for cases with physician contact have not reached the non-inferiority sample size and we did not perform a sample size calculation to demonstrate superiority.

## Conclusions

The TEMS trial demonstrated the non-inferiority of the tele-EMS combined with quality improvement if the tele-EMS physician was involved. The findings suggest a benefit of the tele-EMS for the limited conventional EMS physician resource, supporting further implementation of the tele-EMS as an additional structure in countries with a conventional physician-based EMS. This approach would allow paramedics to be dispatched alone with the possibility to contact a tele-EMS physician at any time for severe conditions, and on-site EMS physicians for acute life-threatening emergencies with the potentially need for manual skills on scene. Further national and international multicentre studies are needed to confirm our results.

## Supplementary Information

**Additional file 1**. Study protocol.

**Additional file 2**. Amendment of thetrial statistical analysis plan.

**Additional file 3**.** eMethods. eResults. eTable 1**. Other Baseline characteristics.** eTable 2**. Frequency of predefined “Special” diagnoses, with physician contact.** eTable 3**. Frequency of main diagnoses with physician contact.** eTable 4**. Other secondary outcomes.

## Data Availability

Any datasets as well as any additional materials will be made available upon approval of formal request to the corresponding author. The request shall be sent to the corresponding author Prof. Rolf Rossaint, email address: rrossaint@ukaachen.de. Additional file 1, Additional file 2, and Additional file 3 to this article can be found online.
