# Supplementary material for: Telemedical support for prehospital emergency medical service in severe emergencies: an open-label randomised non-inferiority clinical trial
Source: Crit Care. 2023 Jun 30;27:256. doi: 10.1186/s13054-023-04545-z (PMC10311733; doi:10.1186/s13054-023-04545-z)
Supplement: Supplementary file 1 — Additional file 1. Study protocol. [file 13054_2023_4545_MOESM1_ESM.docx]

Supplementary materials to

**Telemedical support for prehospital emergency medical service in severe emergencies: *An open-label randomised non-inferiority clinical trial***

Ana Kowark,^1,2^ * Marc Felzen,^1^ * Sebastian Ziemann,^1^ Stephanie Wied,^3^ Michael Czaplik,^1^ Stefan K. Beckers,^1^ Jörg Christian Brokmann,^4^ Ralf-Dieter Hilgers, ^3§^ Rolf Rossaint,^1 * §^ on behalf of the TEMS-study group^¥^

*contributed equally as first authors

^§^contributed equally as last authors

^*^Corresponding author. Email: [rrossaint@ukaachen.de](mailto:rrossaint@ukaachen.de)

^¥^TEMS-Study group. All contributing collaborators are listed in the appendix of the main manuscript.

**Supplement 1. Study protocol.** English translation including protocol changes and dates.

**Telemedical support for pre-hospital Emergency Medical Service (TEMS-trial):**

**study protocol for a randomised controlled trial**

(English translation)

**Translated protocol version 6**

**May 11, 2017**

**Authors**

Ana Stevanovic^1^

E-mail: astevanovic@ukaachen.de

Stefan Kurt Beckers^1, 2^

E-mail: sbeckers@ukaachen.de

Michael Czaplik^1^

E-mail: mczaplik@ukaachen.de

Sebastian Bergrath^1, 2^

E-mail: sbergrath@ukaachen.de

Mark Coburn^1^

E-mail: mcoburn@ukaachen.de

Jörg Christian Brokmann^3^

E-mail: jbrokmann@ukaachen.de

Ralf-Dieter Hilgers^4^

E-mail: rhilgers@ukaachen.de

Rolf Rossaint^1^**^*^**

^1^Department of Anesthesiology, University Hospital RWTH Aachen, Pauwelsstr. 30, 52074 Aachen, Germany

^2^ Emergency Medical Service, Fire Department, Stolberger Str. 155, 52068 Aachen, Germany

^3^Emergency Department, University Hospital Aachen, Pauwelsstr. 30, 52074 Aachen, Germany.

^4^Department of Medical Statistics, University Hospital RWTH Aachen, Pauwelsstr. 30, 52074 Aachen, Germany

| **Originally approved protocol version** | **Date approval by ethics committee; No.** |
| --- | --- |
| Version 3; November 11, 2015 | November 25, 2015; EK 170/15 |

| **Protocol changes** | **Summary of changes** |
| --- | --- |
| Version 4; June 13, 2016 | - 90 days follow-up was added. |
| Version 5; September 2, 2016 | - 1.5-2 months lasting “Run-in” phase was prepended.  - Added retrospective analysis of all EMS cases, which were excluded according to the exclusion criteria and treated by a conventional EMS physician during the study period.  - Extension of the main study period to 8-10 months with the earliest beginning after December 01, 2016.  - Addition that the retrospective analysis is performed pseudonymised and without patient informed consent.  - Addition of Dr. Ana Stevanovic as an investigator. |
| Version 6; May 11, 2017 | - New sample size calculation was added. New sample size comprises 3344 patients.  - “Non-inferiority” wording was specified in the objectives.  - It was specified that the data safety board members receive blinded data, unless the total number of adverse events exceeds 6%.  - The number of needed patients for the first and second interim analysis was adapted according to the new sample size.  - Change of randomisation process into an automatised process, which is implemented in the dispatching software. |

**Abstract**

**Background**

Increasing number of emergency calls, shortages of Emergency Medical Service (EMS) physicians and prolonged emergency response times with delay and regional different quality of treatment by EMS physicians, require an improvement of this system. Telemedical solutions were shown to be beneficial in different emergency projects, focused on specific disease patterns. Our previous pilot studies have shown that the implementation of a holistic pre-hospital EMS teleconsultation system, between paramedics and experienced tele-EMS physicians, is safe and feasible in different emergency situations. We aim to extend the clinical indications for this teleconsultation system. We hypothesise that the use of a tele-EMS physician is non-inferior in regard to the occurrence of system-induced patient adverse events and superior in regard of secondary outcome parameters such as guideline-conform treatment- and documentation-quality when compared to the conventional EMS physician treatment.

**Methods/ design**

3344 patients will be included in this single-centre, open label, randomised controlled, non-inferiority trial with two parallel arms. According to the inclusion criteria, all emergency cases of adult patients, who require an EMS physician treatment, excluding life-threatening cases, will be randomly assigned by the EMS dispatching centre into two groups. 1672 patients in the control-group will be treated by a conventional EMS physician on scene, and 1672 patients in the intervention-group will be treated by paramedics, which are concurrently instructed by the tele-EMS physicians of the teleconsultation centre. The primary outcome measure will include the rate of treatment-specific adverse events depending on the used kind of EMS physician. The secondary outcome measures will record the specific treatment associated quality indicators.

**Discussion**

Evidence underlines better quality of supply with telemedicine networks between medical personnel and medical experts in pre-hospital emergency care, as well as in other medical areas. The worldwide unique EMS teleconsultation system of Aachen was optimised and evaluated in pilot-studies and subsequently integrated in the routine use for a broad spectrum of indications. It enabled prompt, safe and efficient patient treatment with optimised use of the "resource" EMS physician. There is a lack of evidence, whether the advantages of the teleconsultation system could be confirmed in wide-ranging EMS physician indications (excluding life-threatening-emergency calls).

**Trial registration**

ClinicalTrials.gov: NCT02617875, registered November 24, 2015

**Background**

The German and some other European Emergency Medical Services (EMS) consist of a dual system with two paramedics (professional education of two years) and one EMS physician accompanied by a further paramedic. The emergency call is received by the EMS dispatch centre and answered by a specially trained paramedic [1], who dispatches either solely an ambulance vehicle with paramedics or simultaneously, in life-threatening cases, also an EMS-physician vehicle. This is called the rendezvous system. If only an ambulance vehicle was dispatched, but the on-site patient situation requires an EMS physician (e.g. more life-threatening case, or a special medication like opioid analgesia for severe pain management or a drug to reduce systemic arterial pressure in case of severe arterial hypertension is required), then an EMS physician can be demanded by the paramedics on site. The German nationwide network of ambulance vehicle locations shows a significantly higher density than the emergency physician locations [2]. This is due to the fact that the national law and local regulations of the 16 states of Germany define the maximal time frame for the EMS aid in Germany. Hence, the paramedics usually arrive several minutes earlier than the EMS-physician on the emergency scene. The paramedics have to start with the primary emergency medical care on their own authority, but within the restrictions of the German law and local regulations. The time delay until the arrival of the EMS-physician is more pronounced in rural than in urban areas and can jeopardise the patients by omission of physician dependent treatments. Increasing number of emergency calls, of vacant EMS physician sites due to lack of emergency physicians, and increasing loads of the EMS physicians, hinder the disposability of EMS physician treatment [3]. Paramedical teams solely cope 56% of the current emergency calls in Germany, the remaining 44% require an additional EMS physician [2]. This ratio depends on the region and the ratio shifts to more EMS-physician requirements in rural areas in comparison to urban areas. Furthermore, the arrival time of the EMS physician to the emergency scene has become considerably prolonged in 95% of the emergency cases during the last twenty years in Germany [4-6]. This time interval increased from 18.9 to 28.2 minutes in the years 1995 to 2012 and it was more than 20 minutes in 17% of the cases in 2012. The obligatory EMS response time in the German state of North Rhine-Westphalia of 8 minutes for the first EMS unit in urban areas, and 12 minutes in rural areas is based on the recommendations according to the law (RettG NRW). These data reveal that the time interval between the arrival of the EMS ambulance and the EMS physician is frequently prolonged. In addition, as recently shown in the German state of Rhineland-Palatinate, some EMS physician sites do not provide continuous operational readiness due to shortages of physicians [7]. Although there is a lack of systematic data for all German states, it is obvious that similar conditions are throughout present, especially in rural German areas. In these cases EMS physicians from farther away locations or helicopter emergency medical services mostly during the daytime become required. This circumstance further implies prolonged response times of EMS physicians and the solely restricted treatment by paramedics until their arrival. In many cases the paramedics are not able or not allowed to administer adequate aid (i.e. administration of opioids or invasive procedures) [8].

In summary, Germany has a well established ground based EMS, but five problems remain:

- Prolonged emergency response times with delayed treatments by EMS physicians
- Increasing numbers of emergency calls
- Shortages of EMS physicians
- Out-dated communication systems
- Regional different and improvable emergency treatment quality

**Mobile pre-hospital EMS teleconsultation**

Telemedicine networks between medical personnel and medical experts were shown to be beneficial for the quality of supply in many medical fields. National and international teleconsultation is increasingly used for emergency care of stroke patients between hospitals with and without specialised stroke units [9-13]. Hereby it was shown that the real-time use of digital observation cameras was more favorable than the telephone-consultation [12,13]. Furthermore, transmission of a pre-hospital 12-lead electrocardiogram (ECG) and telephone-consultation with a cardiologist improved the emergency treatment and the outcomes of acute myocardial infarction patients [14-18]. Other pre-hospital teleconsultation systems in emergency medicine are rare and were only used in pilot-projects [19,20]. The American Heart Association (AHA) emphasised the use and scientifically evaluation of teleconsultation systems in the pre-hospital emergency care [21-23]. The German research project "Stroke Angel" showed that the use of a tablet computer, with structured collection of stroke-specific patient data and the automatically forwarding to the hospital, decreased the pre-hospital process time of acute stroke management to the half [20]. One disadvantage of this concept is the very specific focus on one medical condition.

The obvious need for a safe and broad usable tele-emergency system stimulated us to develop a holistic multifunctional, algorithm-based, mobile teleconsultation system, as a complementary structural element to the ground based and air based EMS.

**Routine use of the EMS teleconsultation system in Aachen**

After the favorable experience during the two pilot studies [19,24], the EMS teleconsultation system was step-wise introduced in the routinely use, complementary to the ground and helicopter based EMS of Aachen. Since 2014 the costs are covered by the health insurance funds. All ambulance vehicles are equipped with the teleconsultation system since 2015. Up to day the teleconsultation EMS physician (tele-EMS physician) is mainly used for distinct cases such as hypertensive emergency, stroke, dislocated fractures, which are originally dispatched only to the paramedics. The decision to involve a tele-EMS physician is made by the paramedics on scene based on standard operating procedures (SOP) by the EMS medical director, after verbal informed consent of the patient. Additionally, the paramedics decide on their own, whether they demand a physically present EMS physician on scene. Therefore the tele-EMS physician is used either during the time gap until the arrival of the physically present EMS physician or the tele-EMS physician provides medical and organisational advice without a physically present EMS physician. This offers the opportunity for the paramedics to administer medications and perform invasive procedures under the supervision by the tele-EMS physician, which is otherwise not possible by national laws and local regulations. For example paramedics are not allowed to administer opioids without a delegation by a physician. If adverse events (AEs) occur, the tele-EMS physician can give an advice to prevent possible consecutive damages. The teleconsultation system in Aachen protects the patients from delayed physician-dependent treatments and provides a high-quality medical therapy by using software–based guideline conform treatment. A delegation of in-hospital opioid application by physicians to nursing stuff is usual, but unusual in EMS. Severe pain or other threatening conditions require a prompt and accurate medical treatment. In one pilot study, conducted in the EMS of the central State of Hessen, an EMS physician delegated by phone the morphine application by paramedics in 172 patients with limb trauma. This resulted in no threatening AEs and the pain could be significantly reduced [25]. In contrast to the in-hospital delegation by phone and in the latter study, the tele-EMS physician in Aachen monitors (ECG, pulse oxymetry, blood pressure measurement, voice communication with the paramedics) the patient continuously during the treatment until the patient is handed over to a further physician (EMS physician or physician in hospital). This system holds the potential to improve patient safety with online medical control of the paramedics. Concurrently the obligatory indications for dispatching of an EMS physician were confined since March 2015, as a tele-EMS physician is available at any time. This refers for example to the emergency cases, where the decision for drug application is required. Meanwhile 4219 patients (1 April 2014 – 31 March 2016, worldwide the largest case series) were routinely treated with this EMS teleconsultation system in Aachen. Until now, the primary dispatching of the tele-EMS physician was waived. But, analysis of the severity level of the EMS responses by paramedics with the secondary use of the tele-EMS physician shows similar severity levels as the primary dispatched EMS physician staffed responses. Furthermore, the use of the tele-EMS physician instead of a conventional EMS physician did not show any complications and the quality of the medical history survey, the medical treatment and the documentation of "Tracer" diagnoses like stroke and myocardial infarction improved. Also the engagement time of the physician decreased to the half with the use of the teleconsultation EMS. Our experience from the routine use of the teleconsultation EMS system in Aachen - excluding critical emergency cases like severe trauma, cardiopulmonary resuscitation and advanced airway management - shows that this system may provide an equal or even better quality of care than the conventional EMS system. Furthermore, this system enables more economical utilisation of the EMS physician resources. A nationwide implementation of this teleconsultation EMS system in all dual EMS systems requires the proof that it is not causing harm and shows some advantages in cases of primary tele-EMS physician dispatching (excluding life-threatening emergency calls).

**Objectives and study design**

The TEMS trial is designed as a single-centre, prospective, randomised interventional, open label, two-arm parallel group sequential, non-inferiority trial (Fig. 1). The purpose of this trial will be to assess:

- The quality of pre-hospital emergency care using the multifunctional teleconsultation system between paramedics and experienced tele-EMS physicians.

**Specific primary objective**

To determine if the primary usage of a tele-EMS physician compared to a conventional EMS physician on scene, in non-life-threatening cases, is non-inferior with regard to the occurrence of system-induced patient AEs.

**Specific secondary objective**

To determine if the primary usage of a tele-EMS physician compared to a conventional EMS physician on scene, in non-life-threatening cases, is superior in regard to the quality of the medical history survey, medical treatment and documentation.

**Other secondary objectives**

- To determine the different durations between the emergency call, the emergency response by a physician and the arrival time in the hospital.
- To determine the National Advisory Committee for Aeronautics (NACA) score classifications of the treated patients.
- To determine the number of emergency cases, which were handed over to the tele-EMS physician.
- To determine the number of additional requirements of EMS physicians on scene in the case of primary dispatched tele-EMS physicians.
- To determine the technical performance.
- To determine the direct costs involved in the two systems.
- To determine the acceptance of the teleconsultation system by the paramedics, the EMS physicians, the patients and the emergency room personnel.
- Parallel to the randomised main study, we aim to retrospectively analyse all EMS cases, which were excluded according to the exclusion criteria and treated by a conventional EMS physician during the study period.
- To determine the frequency of tele-EMS physician consultation by a conventional EMS physician.
- To analyse additional data, which will be collected during a prepended "Run-In" phase of 1.5-2 months.

**Methods**

**Study setting and design**

This prospective single-centre, randomised interventional, open label, two-arm parallel group sequential trial will be conducted in the urban EMS of Aachen, Germany. This study protocol is reported according to the SPIRIT statement and the SPIRIT checklist is provided in the Additional file 1.

**Eligibility criteria**

**Patient inclusion criteria:** All non-life-threatening emergency calls, which do not obligatory require an EMS physician on scene and which do not solely require an ambulance vehicle staffed with paramedics, will be included in this study. This results in the following case for randomisation:

- An emergency call is received by the EMS dispatching centre Aachen. The EMS dispatching personnel decides after structured interview of the caller, that there is not a life-threatening situation (according to the exclusion criteria), but a physician is required in addition to the paramedics.

**Patient exclusion criteria:**

All life-threatening emergency cases, where a physically present EMS physician on scene is obligatory required according to German regulations. These include patient condition and emergency case related indications:

a) Patient condition related indications:

- Apnoea
- Acute respiratory failure
- Cardio-circulatory arrest
- ST-elevation myocardial infarction (STEMI)
- Unconsciousness
- Persistent seizure
- Life- threatening rhythm disorder
- Major trauma
- Complex psychiatric disorders
- Age < 18 years

b) Emergency case related indications

- Major vehicle accident
- (Traffic) accident with children
- Fall from a height (> 3m)
- Gunshot-, stab-, or blow injuries in the head, neck and torso area
- Fires with reference to personal injury
- Explosion-, thermic or chemical accidents with reference to personal severe injury
- High-voltage electrical accident
- Water connected accidents (drowning-, diving accident, fall through ice)
- Accidents involving hazardous goods
- Hostage-taking, rampage or other crimes with potential danger for human life (preventive deployment, police consultation)
- Immediate threatening suicide
- Immediate forthcoming delivery or preceding delivery

**Requirement of telemedical facilities**

All routinely used EMS ambulance vehicles of Aachen are equipped and connected to a specific teleconsultation centre. This system consists of four sections: The teleconsultation centre, a server infrastructure, a fix-mounted in vehicle PC and a portable autonomous communication device called the “PeeqBOX", as previously described [26,27].

**Teleconsultation centre**

The teleconsultation centre is staffed with experienced EMS physicians from the Department of Anesthesiology of the University Hospital of RWTH, Aachen, Germany and physicians of the P3 Telehealthcare GmbH, Aachen, Germany. During the clinical routine one 24/7 tele-EMS physician is required. In the course of this study an additional tele-EMS physician will be engaged. Minimal educational requirement for tele-EMS physicians comprises a minimal experience of 4 years in anesthesiology and intensive care medicine and an EMS physician certification, furthermore additional qualifications like European Resuscitation Council Provider and Pre-Hospital Trauma Life Support. All tele-EMS physicians have to pass a specific training with regard to the telemedical characteristics including communication principles. The teleconsultation centre is located next to the dispatching centre of the region Aachen. Live vital data (curves and numerical data of ECG rhythms and if needed 12-lead ECG, pulse oxymetry, end-tidal CO_2_ concentrations, blood pressure measurements) are transmitted to the tele-EMS physician. Solely the data transmission systems of the company GS Stemple (Kaufering, Germany), which are approved according to the medical devices law, are used. In addition to this diagnostic data, still picture transmission and video transmission data are available to the tele-EMS physician. These data are explicitly used additionally to the verbal communication with the paramedics and not for determination of diagnosis. The content of this visual data has to be verbally verified. SOP, guidelines and drug databases are IT-based provided to the tele-EMS physician. Standardised, context-sensitive and checklist-based documentation software is used in the teleconsultation centre for all emergency cases conducted with the aid of a tele-EMS physician. Data transfer from the emergency scene, respective the ambulance vehicle to the teleconsultation centre, as well as the bidirectional audio connections are encrypted according to the state of the art. This enables highest degree of interception protection during data transmission. To further improve data safety, all data are marked with an electronic key. This key verifies the authenticity of incoming data in the teleconsultation centre and only accurate data become presented. Data storage occurs on specially secured servers with restricted access, only permitted to the medical project management. Video data are not stored, but only streamed.

**General interventions for all patients**

The patients will be randomised into two groups according to Fig. 1.

1.) Conventional EMS

A physically present conventional EMS physician on scene will treat the patients according to the memorised or carried along written SOP.

2.) Teleconsultation EMS

The patients will be treated by the paramedics, which are concurrently instructed by the tele-EMS physicians of the teleconsultation centre according to the IT-based SOP, guidelines and drug databases.

**Interventions—modifications**

A premature discontinuation of the randomised treatment in a patient is only possible for the teleconsultation EMS group in three situations:

1.) If a relevant technical defect occurs, the teleconsultation will be discontinued. A repair attempt of the teleconsultation system should be omitted during the patient treatment. A conventional EMS physician will be dispatched immediately. A technical repair and diagnosis can follow after termination of the emergency case.

2.) If a patient waives the initially issued informed consent, after the beginning of the teleconsultation treatment, a conventional EMS physician will be dispatched immediately.

3.) If a situation develops within the treatment period which requires a conventional physician according the exclusion criteria, a conventional EMS physician will be dispatched immediately.

In the event of modifications or discontinuations of the study treatment, study participants will be retained in the study to enable data collection and preclude missing data.

**Outcomes**

Participant timeline is shown in Fig. 2.

**Primary Outcome Measures**

The primary outcome measure will include the rate of AEs depending on the specific treatment by an EMS physician or tele-EMS physician. A clinical endpoint committee (CEC) will perform the endpoint adjudication by blinded evaluation and assignment of AEs into intervention-related AEs, according to predefined criteria.

These intervention-related AEs are defined as follows:

- Immediate allergic reaction to drug application due to incorrect survey of patients` medical history (omission of the question for drug allergies).
- Intervention-related and immediate treatment-requiring blood pressure drop on scene (e.g. after wrong drug dosage or drug selection).
- Immediate intervention-related apnoea or respiratory insufficiency on scene (e.g. after wrong drug dosage or drug selection).
- Intervention-related circulatory arrest within 24h of EMS treatment (e.g. after wrong drug dosage, drug selection, or wrong hospital referral).

**Secondary Outcome Measures**

The secondary outcome measures will record the specific treatment associated quality indicators depending on the use of an EMS physician or tele-EMS physician. These specific treatment associated quality indicators are defined as:

- Quality of medical history survey (adherence to the guidelines).
- Treatment quality (adherence to the guidelines).
- Quality of documentation
- Duration of the physician engagement-time.
- Fulfillment of predefined quality indicators for "Tracer" diagnoses
- Trauma
- Stroke
- Acute coronary syndrome
- Pain control
- Bronchial asthma
- Chronic obstructive pulmonary disease (COPD)
- Seizure
- Sepsis
- Hypoglycemia
- Frequency of main diagnoses for all emergency cases
- Correct pre-hospital diagnosis (comparison to the hospital discharge diagnosis)
- AEs (independently of the kind of EMS care, e.g. allergic reaction despite adequate survey of medical history, not-intervention related blood pressure dropping, apnoea or cardiac arrest)
- Other intervention-related AEs
- Premature termination of the telemedical or conventional EMS operation, as unnecessary
- Required conversion from the primary dispatched tele-EMS physician to a conventional EMS physician
- Assessment if a conventional EMS physician operation could have been handled by a tele-EMS physician

**Other Outcome Measures**

The following variables will be assessed in each randomisation group:

- Time point of the first contact with a physician, time span between the emergency call and hospital arrival
- Seven-step National Advisory Committee for Aeronautics (NACA) severity score
- Proportion of conventional emergency cases, which were passed to a tele-EMS physician (differentiated into medical need and lack of capacity)
- Assessment if an EMS physician was necessary at all for each emergency case.
- Technical performance
- Survey of the paramedics, the patients, the EMS physicians and the emergency room personnel in regard to their satisfaction with the used EMS system.
- Death within 24 hours and until day 30 of hospitalisation, respectively until discharge from hospital
- Death within 30 and 90 days after EMS treatment
- Discharge destination from hospital
- Intensive Care Unit (ICU) and hospital length of stay
- Frequency of tele-EMS contacting by a conventional EMS physician for any kind of advice
- Assessment of the medical education/ experience of the involved physicians in each group
- All outcomes will be assessed in addition, for patients which will be recruited during the "Run-In" phase
- Retrospective analysis of the same data (excluding the prospectively collected satisfaction surveys and 30 and 90 day follow-up), like in the main study for the excluded conventional EMS physician cases. These data will be collected from the conventional EMS physician protocols and the hospital database.

**Participant timeline**

A time schedule according to the SPIRIT figure shown in Fig. 2.

**Run-In phase**

1-2 months prior to the main study, we will assess the same outcomes like in the main study.

**Phase A: Enrolment**

The dispatching personnel in the EMS dispatching centre of Aachen will screen all emergency calls for eligibility and enter a suspected diagnosis into the dispatching software.

**Phase B: Allocation**

All non-life-threatening emergency calls, which do not obligatory require an EMS physician on scene, but cannot solely be resolved by the paramedics, will be randomised into the two intervention groups (conventional EMS physician and tele-EMS physician, respectively) automatically by the dispatching software.

**Phase C: During EMS intervention, post-allocation**

Patients will be treated by both kinds of physicians according to the SOP and all operation related data will be documented in a standardised EMS documentation form according to the recommendation of the German Interdisciplinary Society for Intensive and Emergency Medicine (DIVI). As usual in the present routine, the patients in the tele-EMS physician group will be verbally informed about the use of a tele-EMS physician and the teleconsultation system. A written informed consent for study participation shall be obtained as soon as possible during this phase until discharge from hospital for all follow-up visits after the initial EMS intervention.

**Phase D: Early follow up, post-allocation**

A survey of the patients in regard to their satisfaction with the used EMS system should be conducted after hospital arrival until discharge. Assessment of the outcome death within 24 hours and until hospital discharge day, respectively. Assessment of ICU and hospital length of stay and the discharge diagnosis of the hospital.

**Phase E: Late follow up, close-out maximum until day 90**

Additionally, we will assess the mortality within 30 and 90 days, respectively.

**Phase F: Additional analysis, parallel to the main study**

Retrospective analysis of the non-randomised, with conventional EMS-physician treated patients. Satisfaction survey of the paramedics, EMS physicians and emergency room personnel.

**Sample size**

The sample size calculation is based on an assumed AE rate of 2% for the conventional physician based EMS. The rate of 2% is based on our own analysis of 100 EMS physician cases, as we could not find any information about EMS-depending AEs in the literature. We assumed a non-inferiority margin of 1.5% and allocated the overall 5% significance level to K = 3 (power (1 – β) 80.0%). Interim analysis will be performed according to the procedure of O'Brien und Flemming [28]. The critical values, power and sample sizes for the group sequential design are given in Table 1.

Using an allocation ratio of (n2/n1) = 1, the necessary sample size is 1504.2 + 1504.2 = 3008.4, thus resulting in a total sample size of 3010 patients. A fixed sample size design would need n1 = 1478.5 and n2 = 1478.5. The expected total sample size under the alternative is 2531.7. A stop for futility is not planned. Based on an expected dropout rate of 10% we aim to include 3344 patients in total (1.672 per group) in our main study.

**Recruitment**

Beside our main study, we will initially perform a 1.5-2 months "Run-In" phase, to optimise the study conduction processes. During this period, we aim to enroll, randomise and follow-up around 300-600 additional patients, which will be analysed separately from the main study. This will enable process optimisations and increase the compliance of the entire involved personnel. Thereafter, we will recruit 3344 patients, according to the sample size calculation of the main study. Patients will be recruited by the dispatching centre of Aachen. This implies the evaluation of the emergency call severity and exclusion of the life-threatening cases listed in a written procedure instruction. The impact of selection and allocation sequence bias will be determined with the type 1 error.

**Randomisation**

According to technical conditions the randomisation procedure is implemented in the software of the dispatching centre. The software is provided by the company ISE GmbH, Schönebergstr. 15, 52068 Aachen, Germany. Therefore, other than initially planned, ISE and the Department of Anaesthesiology are responsible for randomisation including assessment of appropriate procedure. Details of the randomisation procedure will be described in a randomisation report, which will be kept concealed until closure of the database. The randomisation sequence will be implemented into the dispatching software and will therefore remain concealed until all important data including the suspected diagnosis of the next emergency case are entered into this system by the dispatching personnel. Following, the dispatching software will automatically randomise all patients with eligible suspected diagnoses into the two intervention groups. Hereinafter the dispatching personnel in the dispatching centre will assign the patients according to the randomisation proposal of the dispatching software.

**Blinding**

Blinding of the dispatching personnel, the paramedics on scene, patients and the physicians is not possible due to practicability reasons. Furthermore the electronic case report form (e-CRF) entering personnel cannot be blinded. The outcome assessors of the late follow-up variables and the CEC for the primary endpoint adjudication will be blinded to the kind of EMS intervention. The data safety monitoring committee (DSMC) will primarily be blinded. Only if the total number of all AEs exceeds 6%, the DSCM will not be blinded due to safety reasons.

**Un-blinding procedures**

There are no events expected to un-blind the outcome assessors.

**Data collection methods**

The entire EMS personnel (including the dispatching personnel, the physicians and the paramedics on scene) will be informed about the study procedures and supervised by the principal investigator. The principal investigator will ensure local training of the entire EMS personnel to enhance the data collection quality and reduce bias. Furthermore, the assisting study personnel will have to be adequately qualified and informed about the study protocol, any amendments, and study related responsibilities and functions. A study staff authorisation log will be maintained. Every reasonable effort will be made to follow each enrolled and randomised patient until completion of study phase E.

**Participant withdrawal and loss to follow up**

Participants may withdraw from the study for any reason at any time. The follow-up period in this study is maximum 90 days. We do not expect many withdrawals or losses to follow up until phase E. For phase E we expect a higher loss to follow up data for the outcome variable mortality on day 90. The reasons for missing data will be recorded.

**fData management**

SOP for data management will be implemented to ensure accurate, consistent, complete and reliable data. All collected data from a subject during the course of this trial have to be filled and/ or entered in the respective patient study case report form (CRF).

As source data will serve:

- The standardised routinely created EMS files on scene (during both the conventional physician as well as the tele-EMS physician operation).
- The additional EMS documentation files created in the teleconsultation centre (including the transmitted patient vital data, e.g. ECG and blood pressure measurements).
- The routinely conducted EMS operation time recording in the dispatching centre.
- Data from the admission hospital database: Diagnoses according to the 10th version of the International Classification of Diseases (ICD 10), time-points of the relevant diagnostic or therapeutic procedures (e.g. computer tomography, cardiac catheter examination), laboratory data and severity scores of diseases (e.g. National Institutes of Health Stroke Scale for stroke patients).
- The clinical follow up data like death after 24h, or 30 respectively 90 days; the ICU and hospital length of stay and the hospital discharge diagnosis, which are already collected for quality assurance reasons, based on the Law on Rescue Services of the state of North Rhine-Westphalia (RettG NRW, version of 18 March 2015); and the hospital discharge destination, will be collected from the hospital database.

A high validity of the collected data can be assumed, as they are collected during in the clinical routine and not specifically for the study. Furthermore there will not be an additional burden for the medical personnel and any acquisition and storage of sensitive and confidential patient data, as all data will be entered pseudonymised into the CRF. According to a SOP all relevant source data will be manually entered by the study personnel in a validated e-CRF form. The respective patients` paper-based CRF will contain the written informed consent with the date of subject information, a unique study and subject number.

Paper printouts of study data from an electronic database have to be signed and dated by a member of the site staff, to confirm the accuracy and completeness of data in the paper printout. Additionally, the monitor shall sign and date the verified paper printout. The paper printout shall be stored in CRF. Retrospectively entered source data information on this paper printouts have to be initiated and dated. Furthermore, any corrections of the original data require to draw a single line through the error and to sign with the date and the initials. Hereby, the crossed out entry should remain legible. The investigator shall not falsify the data.

**Clinical Endpoint Committee**

Blinded CEC members will perform the primary endpoint adjudication, to enhance the validity of the assignment of the four pre-specified AEs into intervention-related or not. The committee will consist of three members, which are fully independent from the investigators and the sponsor.

**Statistical methods**

To prove the non-inferiority hypothesis that the system-induced patient AE rate by the treatment of the tele-EMS physician compared to the conventional EMS physician is not inferior by 1.5% of the (1-α)% confidence interval for rate differences will be calculated accounted for the interim analysis (Table 1). The hybrid analysis population as developed by Sanchez will be used as analysis population [29]. Further details will be given in the trial statistical analysis plan, before database lock. The secondary dichotomous parameters will be tested by chi-square test, the continuous parameters by t-Test assuming heterogeneous variances. A subgroup analysis will be performed according to the level of medical education/ experience of the involved physicians in each group.

**Data monitoring**

This study will be conducted in accordance with the approved protocol version, the ICH-GCP (International Conference on Harmonisation of Technical Requirements for Registration of Pharmaceuticals for Human Use-good clinical practice) principles, the Declaration of Helsinki, regulatory authority requirements, and the SOP.

Qualified monitors from the sponsor Clinical Trial Centre Aachen (CTC-A) will perform the monitoring visits according to the ICH-GCP principles and their SOP. Before the initiation of this study there will be one or more monitoring visits, to check and clarify the prerequisites. The investigator is obliged to enable direct access to the source data and CRFs for study specific monitoring, auditing and inspections by the competent ethics committee. He/ she will support the respective person and be available for questions. Regular monitoring visits of random samples of data should discover and remove grievances and verify that the data collection and documentation process is conducted in concordance with the ICH-GCP principles, the study protocol and the regulatory authority requirements. The following processes and data will be reviewed in a random sample of data during the monitoring visits:

1. Entries in the e-CRF
2. Adherence to the study protocol, the ICH-GCP principles, the Declaration of Helsinki, and the regulatory authority requirements
3. Integrity of the source data and the e-CRF entries
4. Accuracy of the documentation and report of AEs and serious adverse events (SAEs) within the required time periods.
5. Patient identification, screening and enrollment log, study staff log and the monitoring log
6. Accuracy and completeness of the trial master file

Further monitoring details will be set forth in the monitoring manual.

**Data safety monitoring committee and interim analyses**

A formal DSMC will consist of three members with no competing interests and fully independent from the sponsor and investigators. Comprehensive analyses of the documented adverse events will be performed on monthly basis. They will be assessed on specific forms. The frequency of the pre-defined AEs (please see primary outcome measures) as well as non-intervention-related AEs (please see secondary outcome measures) and resuscitation within 24h of hospital admission will be referred to the safety board committee. This committee will assign, if the AEs are intervention-related or not. If there appears a difference of 5% intervention-related AEs in one study-arm, then the safety board has to recommend a prematurely study termination. Due to the complexity of the data, a useful interim analysis is planned after inclusion of 1.115 and 2.229 patients, in regard to the primary outcome variable. But if the analysis of the AEs indicates the need for an interim analysis, this will be conducted immediately.

**Harms**

We expect an increase of patient safety with the support of a tele-EMS physician in emergency cases. An intensive standardised training of the entire EMS personnel in regard to the teleconsultation system was performed already before the implementation in the routinely use. This training focused on the transmission technology and the uniform and purposeful communication. This should enable a structured, efficient and safe transmission of the conversation content. Furthermore, legal matters like the informed consent of patients, the legal relationship between the paramedics and the tele-EMS physicians and liability risks were discussed and clarified. A further safety measure is the support of the tele-EMS physicians by the software-based checklists. The advantages for checklist-based operations were shown for the patient safety within the WHO project: "Safe Surgery Saves Lifes". An improved patient outcome was shown for elective as well as emergency surgery with the checklist-based working [30-32]. The SOP for the teleconsultation system defines such checklists for the tele-EMS physicians, to ensure a necessary minimum standard of teleconsultation. E.g. intravenous drug application is only permitted if all required information is collected and all safety measures are done (like pulse oxymetry, blood pressure measurement, ECG rhythm analysis). The tele-EMS physician evaluates the validity and reliability of the teleconsultation data transfer. In cases, with low rates of data transfer and interruptions of a continuous patent monitoring, any delegation to the paramedics is not allowed according to the SOP, if a continuous monitoring is medically indicated. However, as actually nationwide usual, the paramedics would further on treat the patients, even in the case of a complete interruption of the teleconsultation system. All paramedics are educated and trained in advanced life support. Furthermore, a back up mobile phone is available in each ambulance vehicle, in accordance with German industrial standard (DIN EN 1789) and the teleconsultation centre is provided with a fixed-line connection in case of an interruption of the Internet connection. An IT-safety concept was established in collaboration with the data protection officer of the city of Aachen, to protect all transmitted and collected data against unauthorised access, unauthorised modifications and data loss. This IT-safety concept comprises also the storage time frame and the access rights.

AEs are possible in regard to technical, organisational or medical factors. Occurrence of AEs during the teleconsultation has to be documented in standardised forms provided to the tele-EMS physicians and the paramedics. These documentations have to be analysed immediately by the investigators, to recognise problems and potential hazards without time delay and to initiate countermeasures as soon as possible. AEs listed under primary outcome measures are expected in connection with the specific kind of EMS care (conventional / telemedical).

**Definition of AEs**

All unexpected AEs and the expected primary outcome AEs in the course of the EMS treatment have to be documented on an AE reporting form, which will be provided by the sponsor. All investigators will receive an intensive training for the definition, documentation and reporting of AEs by the sponsor. Each AE has to be specified as follows:

1. Duration

2. Severity (mild, moderate, or severe)

3. Causal Relationship to the treatment (suspected/ not suspected)

4. Required treatment of AE and action taken with trial drug

5. Outcome

6. Seriousness

**Definition of SAE`s**

A SAE is an AE which:

1. Results in death (fatal)
2. Is immediately life- threatening
3. Results in persistent or significant disability/incapacity
4. Requires or prolongs patient hospitalisation
5. Results in a congenital anomaly/ birth defect
6. May jeopardise the patient and may require medical or surgical intervention to prevent one of the aforementioned outcomes (for example, intensive treatment in an emergency room without hospitalisation)

SAEs have to be reported by the principal investigator to the sponsor and the DSMC within 24h after detection. All SAEs will be summarised in the annual safety report of the sponsor.

**Ethics and dissemination**

**Research ethics approval**

The study was presented to the Ethics Committee of the University of RWTH Aachen, 52074 Aachen, Germany. The approval with the reference number EK 170/15 was initially received on 23 November 2015. Protocol changes were approved on 20 September 2016. Any further changes of the study protocol, excluding changes for logistic and administration reasons or preventions of immediate hazards have to be approved by the Ethics Committee before their implementation.

**Protocol amendments**

Any amendments, which may affect:

- The patient safety
- The integrity and credibility of data
- Changes in risk evaluation

Every substantial amendment will be submitted, after signature of the principle investigator, the sponsor and the biostatistician, to the Ethics Committee for approval.

**Confidentiality**

All included patients will be pseudonymised. A subject identification log will be managed according to the SOP of the sponsor and safely stored in the investigator’s site file. The investigators will collect all non-anonymised source data (e.g. the EMS forms and the hospital database) and enter them pseudonymised manually in the e-CRF database. This data will only be handed over to a third party anonymised. The Department for Medical Statistics of the University Hospital RWTH Aachen will perform the statistical analysis of the anonymised data. All source data and documents will be stored for 10 years in locked cabinets in the Department of Anesthesiology of the University Hospital RWTH Aachen, with restricted access. Only the study team, and in connection with them also monitors, auditors or the competent Ethics Committee will have access to personal data. In case of a patient withdraw from study, only the anonymised data will be analysed and stored. All regular (not study specific) electronic patient data, which are used for documentation in the clinical routine, are stored on particularly safe servers and stored according to the legal requirements.

**Dissemination policy**

The main results of this study will be published in a leading international journal. Further results of this study will be published in other professional English-speaking journals. The final report will be conducted in concordance with the CONSORT guideline as well as the extension to non-inferiority trials. The study is already registered in the ClinicalTrials.gov registry (NCT02617875).

**Discussion**

Increasing number of emergency calls, prolonged emergency response times by EMS physicians, and shortages of EMS physicians have a big impact on the German EMS system. Furthermore, the EMS treatment [33,34], and documentation quality [35,36] require an urgent improvement.

Evidence was shown for the better quality of supply with telemedicine networks between medical personnel and medical experts in the pre-hospital emergency care as well as in other medical areas. The worldwide unique EMS teleconsultation system of Aachen was optimised and evaluated between 2009 and 2013 in nearly 1,000 patient cases. The system appeared functional and was highly appreciated by the paramedics in Aachen [37]. In the majority of cases it reduced the requirement of EMS physicians and on the other hand it enabled medical advices by EMS physicians to the paramedics, without time delay. The project "TemRas" showed that an EMS teleconsultation system with a trans-regional teleconsultation centre is feasible, can be operated without any complications, and which requirements are demanded for its implication in the daily use [7,24]. Transfer of real time patient vital data, pictures and the audio communication system function between the paramedics on scene and the physician in the teleconsultation centre were fast and reliable [26,27,38,39]. This enabled a prompt, safe and efficient patient treatment, even under unavailability of a physician on scene or until the arrival of the EMS physician. It enhanced the optimised use of the "resource" EMS physician. After its evaluation, it was implemented in the routine use of the EMS system in Aachen. All conducted emergency missions with the aid of the tele-EMS physician were uneventful and provided a better quality of the medical history survey, the treatment and the documentation. Furthermore, the physician engagement time was shortened to the half. Until now, this system was only used for the primary dispatched ambulance vehicles staffed with paramedics, with restricted EMS physician indications.

The need of EMS physicians for emergency missions increases about 2-3% per year since 20 years in Aachen. Before the implementation of the multifunctional mobile teleconsultation system an EMS physician was needed in 34-36% of the emergency missions, now it deceased to about 26%, which is considerably less frequent than in the national average [40].

There is a lack of evidence, if the advantages of the teleconsultation system instead of a physically present physician on scene, could also be confirmed in further cases with EMS physician indications (excluding life-threatening-emergency calls).

We would like to acknowledge some potential bias in our study: Different levels of medical education or EMS experience within our physicians cannot be excluded during our study conduction in the clinical routine. Especially, our minimal educational requirement for the tele-EMS physician is beyond the minimal educational requirement for the conventional EMS physician, according to the guideline of the General Medical Council of North Rhine-Westphalia. On the other hand, we have also conventional EMS physicians, with much more experience than our minimal educational requirement for the tele-EMS physician. This potential bias will be addressed in our statistical analysis with data presentation on the level of medical education/ experience of our involved physicians in each group. Furthermore, the conventional EMS physician has always the opportunity to contact the tele-EMS physician for any advice. This performance bias will also be addressed in our data analysis.

**Risk benefit assessment**

So far, our experience with the teleconsultation EMS system shows at least an equivalent diagnosis and treatment quality between the tele-EMS physician and the conventional physically present EMS physician on scene. As immediate life-threatening emergency cases (as described in the exclusion criteria) are excluded from this study, a specific risk is neither with the conventional treatment nor with the telemedical treatment expected.

**Trial status**

Patient recruitment is expected to start March 2017. The predicted study recruiting end-date is March 2018.

**List of abbreviations**

AE, adverse event; AHA, American Heart Association; CEC, Clinical endpoint committee; COPD, Chronic obstructive pulmonary disease; CRF, case report form; CTC-A, Clinical Trial Centre Aachen; DFG, German Research Foundation; DIN, German industrial standard; DIVI, German Interdisciplinary Society for Intensive and Emergency Medicine; DSMC, Data safety monitoring committee; ECG, electrocardiogram; e-CRF, electronic case report form; e.g., for example; EMS, Emergency Medical Service; h, hours; ICD 10, International Classification of Diseases 10; ICH-GCP, International Conference on Harmonisation of Technical Requirements for Registration of Pharmaceuticals for Human Use-good clinical practice; ICU, Intensive Care Unit; IT, Information technology; RettG NRW, Law on Rescue Services of the state of North Rhine-Westphalia; SAE, serious adverse event; SOP, standard operating procedures; STEMI, ST-elevation myocardial infarction; StGB, German Penal Code; tele-EMS physician, teleconsultation EMS physician.

**Declarations**

**Ethical approval and consent to participate**

The Ethics Committee of the University of RWTH Aachen, 52074 Aachen, Germany, approved this study. Reference number EK 170/15.

**Consent**

The patient information and consent in regard to the routine use of the teleconsultation system, including the data transfer are already legally regulated in the EMS system of Aachen. This regulation refers to the § 203 subsection 1 No. 1 und subsection 2 No. 1 of the German Penal Code (StGB).

**The routinely informed consent procedure for teleconsultation is defined as follows:**

If the patient is legally competent and there is no emergency situation, the patient has to be verbally informed and a verbal consent into the telemedical procedure has to be obtained. In emergency situations, where the patient is not legally competent, e.g. shock, strong pain condition or impaired consciousness, the consent is justified under the «presumed consent» principle. This principle refers also to legally competent patients, in urgent cases with the aim to prevent serious patient damage. The standardised patient information by the paramedics and the verbal patient consent have to be documented on the EMS form on scene by the paramedics. In addition, the tele-EMS physician has to ensure this consent procedure before the commencement of the telemedical consultation and to document it also on the tele-EMS physician form. In cases, where the patient is not willing to be treated by a tele-EMS physician, the paramedics have to document this and to decide, if they need a conventional EMS physician on scene, or to carry out the EMS operation on their own responsibility.

**The study specific informed consent procedure**

In accordance with the ethical approval, seeking consent for the randomisation procedure and the assigned kind of EMS-treatment is not feasible, as it occurs already before the first contact to a physician. Therefore, consent will be waived for this first step of the study. The EMS-files in both treatment groups and the hospital information system-based outcomes such as the diagnoses and the hospital- or intensive care unit length of stay, will be analysed pseudonymised within the scope of data quality assurance in the EMS of Aachen. A written informed consent is mandatory for the follow-up procedures, patient satisfaction survey, 30- and 90-day follow-up. Further, according to the ethical approval, an informed consent will be waived for the patients, which will be pseudonymised included in the parallel conducted retrospective analysis of the non-randomised conventional EMS physician cases.

**The study specific informed consent procedure for the follow-up procedures in the teleconsultation treatment group is defined as follows:**

1. Scenario: The legally competent patient will be informed about the study and the pseudonymised analysis of his data. This will be performed by trained paramedics on scene (phase C), if the emergency condition allows it (e.g. no life-threatening danger, patient is legally competent). In addition, each patient will receive written patient information with contact data of the principle investigator, where he can receive further information. As soon as possible (phase D), a sub-investigator will personally explain all aspects of the study and the requirements concerning data collection, analysis and protection. All arising questions have to be answered to complete satisfaction of the patient. The patients will be asked to voluntarily confirm their willingness to participate in the study. An informed consent, including the disclosure of individual data has to be signed and dated by the patient and the sub-investigator. A copy of the informed consent form will be provided to the patients.

2. Scenario: For not legally competent patients (e.g. shock, strong pain condition or impaired consciousness) the consent will be justified under the «presumed consent» principle, after individual assessment of the entire situation during phase C. Afterwards, the patients (after regain of legal competency) or the legal representative will be personally informed about the study and the consent will be obtained by the sub-investigator like in the first scenario (phase D).

**The study specific informed consent procedure for the follow-up procedures in the conventional EMS treatment group is defined as follows:**

This procedure will take place in the same manner as described for the telemedical treatment, with one exception: A conventional physician, instead of the paramedic will inform the patients about the study and an informed consent could also be obtained during the phase C of the study.

**Survey of the personnel**

Satisfaction-survey of the paramedics, the EMS physicians and the emergency room personnel in regard to the used EMS system, will be performed with anonymised questionnaires after the EMS operation. Therefore an informed consent can be waived for these surveys.

**Table 1.** Statistical calculation based on Farrington und Manning formula [41].

| Information rate | Bounds accept H0 | Bounds reject H0 | Significance level one-sided | α spent | β spent | Power achieved | Stage n1 | Sizes n2 |
| --- | --- | --- | --- | --- | --- | --- | --- | --- |
| 0.333 | - | 3.471 | 0.0003 | 0.0003 | - | 0.0329 | 501.4 | 501.4 |
| 0.667 | - | 2.454 | 0.0071 | 0.0072 | - | 0.4424 | 501.4 | 501.4 |
| 1.0 | 2.004 | 2.004 | 0.0225 | 0.0250 | - | 0.8000 | 501.4 | 501.4 |

H0 = null hypothesis; n = number

**Literature**

1. Roessler M, Zuzan O. EMS systems in Germany. Resuscitation. 2006;68:45-9.
2. Leistungen des Rettungsdienstes 2012/2013. http://www.bast.de/DE/Publikationen/Foko/Downloads/2015-15.pdf?__blob=publicationFile&v=3. Accessed 20 March 2016.
3. Reimann B, Maier BC, Lott R, and Konrad F. Gefährdung der Notarztversorgung im ländlichen Gebiet. Notfall & Rettungsmedizin. 2004;7:200-4.
4. Behrendt H, Schmiedel R, and Auerbach K. Überblick über die Leistungen des Rettungsdienstes in der Bundesrepublik Deutschland im Zeitraum 2004/05 Notfall + Rettungsmedizin. 2009;12:383-8.
5. Bericht über Maßnahmen auf dem Gebiet der Unfallverhütung im Straßenverkehr 2012 und 2013. http://dip21.bundestag.de/dip21/btd/18/024/1802420.pdf. Accessed 22 February 2016.
6. Schmiedel R, Behrendt H. Leistungen des Rettungsdienstes 2008/09. Analyse des Leistungsniveaus im Rettungsdienst für die Jahre 2008 und 2009. http://www.bast.de/DE/Publikationen/Berichte/unterreihe-m/2011-2010/m217.html. Accessed 20 February 2016.
7. Luiz T, van Lengen RH, Wickenkamp A, Kranz T, and Madler C. [Operational availability of ground-based emergency medical services in Rheinland-Palatinate: state-wide web-based system for collation, display and analysis]. Anaesthesist. 2011;60:421-6.
8. Notfall: Notkompetenz II. http://www.bundesaerztekammer.de/richtlinien/empfehlungenstellungnahmen/notfall-notkompetenz-ii/. Accessed 23 March 2016.
9. Audebert HJ, Kukla C, Clarmann von Claranau S, Kühn J, Vatankhah B, Schenkel J, et al.. Telemedicine for safe and extended use of thrombolysis in stroke: the Telemedic Pilot Project for Integrative Stroke Care (TEMPiS) in Bavaria. Stroke. 2005;36:287-91.
10. Audebert HJ, Schenkel J, Heuschmann PU, Bogdahn U, Haberl RL, and Telemedic Pilot Project for Integrative Stroke Care Group. Effects of the implementation of a telemedical stroke network: the Telemedic Pilot Project for Integrative Stroke Care (TEMPiS) in Bavaria, Germany. Lancet Neurol. 2006;5:742-8.
11. Audebert HJ, Schultes K, Tietz V, Heuschmann PU, Bogdahn U, Haberl RL, et al.. Long-term effects of specialized stroke care with telemedicine support in community hospitals on behalf of the Telemedical Project for Integrative Stroke Care (TEMPiS). Stroke. 2009;40:902-8.
12. Demaerschalk BM, Bobrow BJ, Raman R, Kiernan T-EJ, Aguilar MI, Ingall TJ, et al.. Stroke team remote evaluation using a digital observation camera in Arizona: the initial mayo clinic experience trial. Stroke. 2010;41:1251-8.
13. Meyer BC, Raman R, Hemmen T, Obler R, Zivin JA, Rao R, et al.. Efficacy of site-independent telemedicine in the STRokE DOC trial: a randomised, blinded, prospective study. Lancet Neurol. 2008;7:787-95.
14. Dhruva VN, Abdelhadi SI, Anis A, Gluckman W, Hom D, Dougan W, et al.. ST-Segment Analysis Using Wireless Technology in Acute Myocardial Infarction (STAT-MI) trial. J Am Coll Cardiol. 2007;50:509-13.
15. Adams GL, Campbell PT, Adams JM, Strauss DG, Wall K, Patterson J, et al.. Effectiveness of prehospital wireless transmission of electrocardiograms to a cardiologist via hand-held device for patients with acute myocardial infarction (from the Timely Intervention in Myocardial Emergency, NorthEast Experience [TIME-NE]). Am J Cardiol. 2006;98:1160-4.
16. Sanchez-Ross M, Oghlakian G, Maher J, Patel B, Mazza V, Hom D, et al.. The STAT-MI (ST-Segment Analysis Using Wireless Technology in Acute Myocardial Infarction) trial improves outcomes. JACC Cardiovasc Interv. 2011;4:222-7.
17. Terkelsen CJ, Nørgaard BL, Lassen JF, Gerdes JC, Ankersen JP, Rømer F, et al.. Telemedicine used for remote prehospital diagnosing in patients suspected of acute myocardial infarction. J Intern Med. 2002;252:412-20.
18. Sejersten M, Sillesen M, Hansen PR, Nielsen SL, Nielsen H, Trautner S, et al.. Effect on treatment delay of prehospital teletransmission of 12-lead electrocardiogram to a cardiologist for immediate triage and direct referral of patients with ST-segment elevation acute myocardial infarction to primary percutaneous coronary intervention. Am J Cardiol. 2008;101:941-6.
19. Skorning M, Bergrath S, Rörtgen D, Brokmann JC, Beckers SK, Protogerakis M, et al.. [E-health in emergency medicine - the research project Med-on-@ix]. Anaesthesist. 2009;58:285-92.
20. Ziegler V, Rashid A, Müller-Gorchs M, Kippnich U, Hiermann E, Kögerl C, et al.. [Mobile computing systems in preclinical care of stroke. Results of the Stroke Angel initiative within the BMBF project PerCoMed]. Anaesthesist. 2008;57:677-85.
21. Ting HH, Krumholz HM, Bradley EH, Cone DC, Curtis JP, Drew BJ, et al.. Implementation and integration of prehospital ECGs into systems of care for acute coronary syndrome: a scientific statement from the American Heart Association Interdisciplinary Council on Quality of Care and Outcomes Research, Emergency Cardiovascular Care Committee, Council on Cardiovascular Nursing, and Council on Clinical Cardiology. Circulation. 2008;118:1066-79.
22. Schwamm LH, Audebert HJ, Amarenco P, Chumbler NR, Frankel MR, George MG, et al.. Recommendations for the implementation of telemedicine within stroke systems of care: a policy statement from the American Heart Association. Stroke. 2009;40:2635-60.
23. Schwamm LH, Holloway RG, Amarenco P, Audebert HJ, Bakas T, Chumbler NR, et al.. A review of the evidence for the use of telemedicine within stroke systems of care: a scientific statement from the American Heart Association/American Stroke Association. Stroke. 2009;40:2616-34.
24. Brokmann JC, Rossaint R, Bergrath S, Valentin B, Beckers SK, Hirsch F, et al. [Potential and effectiveness of a telemedical rescue assistance system. Prospective observational study on implementation in emergency medicine]. Anaesthesist. 2015;64:438-45.
25. Greb I, Wranze E, Hartmann H, Wulf H, and Kill C. Analgesie beim Extremitätentrauma durch Rettungsfachpersonal Notfall+ Rettungsmedizin. 2011;14:135-42.
26. Bergrath S, Czaplik M, Rossaint R, Hirsch F, Beckers SK, Valentin B, et al.. Implementation phase of a multicentre prehospital telemedicine system to support paramedics: feasibility and possible limitations. Scand J Trauma Resusc Emerg Med. 2013;21:54.
27. Czaplik M, Bergrath S, Rossaint R, Thelen S, Brodziak T, Valentin B, et al. Employment of telemedicine in emergency medicine. Clinical requirement analysis, system development and first test results. Methods Inf Med. 2014;53:99-107.
28. O'Brien PC, and Fleming TR. A multiple testing procedure for clinical trials. Biometrics. 1979;35:549-56.
29. Matilde Sanchez M, and Chen X. Choosing the analysis population in non-inferiority studies: per protocol or intent-to-treat. Stat Med. 2006;25:1169-81.
30. Haynes AB, Weiser TG, Berry WR, Lipsitz SR, Breizat A-HS, Dellinger EP, et al. Changes in safety attitude and relationship to decreased postoperative morbidity and mortality following implementation of a checklist-based surgical safety intervention. BMJ Qual Saf. 2011;20:102-7.
31. Haynes AB, Weiser TG, Berry WR, Lipsitz SR, Breizat A-HS, Dellinger EP, et al. A surgical safety checklist to reduce morbidity and mortality in a global population. N Engl J Med. 2009;360:491-9.
32. Weiser TG, Haynes AB, Dziekan G, Berry WR, Lipsitz SR, Gawande AA, et al. Effect of a 19-item surgical safety checklist during urgent operations in a global patient population. Ann Surg. 2010;251:976-80.
33. Timmermann A, Russo SG, Eich C, Roessler M, Braun U, Rosenblatt WH, et al. The out-of-hospital esophageal and endobronchial intubations performed by emergency physicians. Anesth Analg. 2007;104:619-23.
34. Qualitätssicherung im Rettungsdienst Baden-Württemberg - Downloads. http://www.sqrbw.de/90.php. Accessed 2 May 2016
35. Bergrath S, Rörtgen D, Skorning M, Fischermann H, Beckers SK, Mutscher C, et al. [Emergency mission documentation in simulated care. Video-based error analysis]. Anaesthesist. 2011;60:221-9.
36. Bergrath S, Skorning M, Rörtgen D, Beckers SK, Brokmann JC, Mutscher C, et al. Is paper-based documentation in an emergency medical service adequate for retrospective scientific analysis? An evaluation of a physician-run service. Emerg Med J. 2011;28:320-4.
37. Czaplik M, Bergrath S, Rossaint R, Thelen S, Brodziak T, Valentin B, et al. Employment of telemedicine in emergency medicine. Clinical requirement analysis, system development and first test results. Methods Inf Med. 2014;53:99-107.
38. Bergrath S, Reich A, Rossaint R, Rörtgen D, Gerber J, Fischermann H, et al.. Feasibility of prehospital teleconsultation in acute stroke--a pilot study in clinical routine. PLoS One. 2012;7:e36796.
39. Bergrath S, Rörtgen D, Rossaint R, Beckers SK, Fischermann H, Brokmann JC, et al. Technical and organisational feasibility of a multifunctional telemedicine system in an emergency medical service - an observational study. J Telemed Telecare. 2011;17:371-7.
40. www.aachen.de - Rettungsdienstbedarfsplan 2014 der Stadt Aachen. http://www.aachen.de/DE/stadt_buerger/politik_verwaltung/feuerwehr/downloads/rettungsdienst/rettungsdienst_2014/index.html. Accessed 20 May 2016
41. Farrington CP, and Manning G. Test statistics and sample size formulae for comparative binomial trials with null hypothesis of non-zero risk difference or non-unity relative risk Statistics in medicine. 1990;9:1447-54.

**Figures**

**Fig. 1 Study design.**





Flow chart of the study conduction in the clinical routine.

**Fig. 2** **Participant timeline SPIRIT Figure.**

Schedule of enrolment, interventions and assessments.

*Specific phases: Run-In phase (1.5-2 months prior study start), A = Enrolment, B = Allocation, C = During EMS intervention, D = Early follow up, E = Late follow up, F = Additional analysis

**Specific time-points: ***-t_2_*** = Run-In phase (1.5-2 months prior study start), ***-t_1_*** = Enrolment during the emergency call in the dispatching centre, ***0*** = Allocation via automatic randomisation by the dispatching software, ***t_1_*** = EMS operation on scene, ***t_2_*** = 24 hours after EMS intervention, ***t_3_*** = Discharge from hospital, ***t_4_*** = Follow-up 30 days after EMS intervention, ***t_5_*** = Follow up 90 days after EMS intervention, ***t_x_*** = Parallel to the main study.
