# Supplementary material for: Telemedical support for prehospital emergency medical service in severe emergencies: an open-label randomised non-inferiority clinical trial
Source: Crit Care. 2023 Jun 30;27:256. doi: 10.1186/s13054-023-04545-z (PMC10311733; doi:10.1186/s13054-023-04545-z)
Supplement: Supplementary file 2 — Additional file 2. Amendment of thetrial statistical analysis plan. [file 13054_2023_4545_MOESM2_ESM.pdf]

AMENDMENT OF THE

TRIAL STATISTICAL ANALYSIS PLAN

TELEMEDICAL SUPPORT FOR PREHOSPITAL EMERGENCY MEDICAL SERVICE – A PROSPECTIVE RANDOMISED CONTROLLED TRIAL (TEMS-TRIAL):

AMENDMENT VERSION 1.0, 09.03.2022

OF TSAP VERSION 1.0, 07.07.2020

Sponsor	RWTH Aachen
Clinical Trials.gov	NCT02617875
Principal Investigator<br>(gem §4 Abs. 25 AMG)	Univ. Prof. Dr. med. Rolf Rossaint<br>Department of Anaesthesiology<br>Medical Faculty, RWTH Aachen University<br>Pauwelsstr. 30,<br>52074 Aachen<br>Germany
Biostatistician	Univ. Prof. Dr. rer. nat. Ralf-Dieter Hilgers<br>Department of Medical Statistics<br>Medical Faculty, RWTH Aachen University<br>Pauwelsstr. 19,<br>52074 Aachen<br>Germany

SUMMARY

1. DESCRIPTION AND RATIONALE OF THE AMENDMENTS 3
1.1 DEFINITION OF THE PRIMARY ENDPOINT 3
1.2 DEFINITION OF SECONDARY ENDPOINTS AND OTHER OUTCOME MEASURES 4
1.2.1 OTHER INTERVENTION-RELATED ADVERSE EVENT 4
1.2.2 QUALITY OF DOCUMENTATION... 4
1.2.3 DURATION OF PHYSICIAN ENGAGEMENT-TIME 5
1.2.4 TIME SPAN UNTIL HOSPITAL ARRIVAL... 5
1.3 MINOR CHANGES.. 5
2. SIGNATURES 7

1. DESCRIPTION AND RATIONALE OF THE AMENDMENTS

1.1 DEFINITION OF THE PRIMARY ENDPOINT

The primary endpoint is defined by a status variable. The status variable is 1 if the patient experiences at least one intervention-related AE and 0 if the patient experiences no intervention related AE. The variables for the assessment of causality in the database have the 5 attributes “not confirmed”, “certain”, “probable”, “improbable”, “no relation”. The evaluation of the AEs of the clinical endpoint committee was judged by the CEC by the attributes “suspected” or “not suspected”. According to the initial version of the TSAP, an AE was only considered to have a sufficient causal relationship if "certain" was ticked in the database. As the attribute " suspected" was not available, we had to change the definition of an AE to be "suspected" of having a causal relationship if either the attribute "certain" or "probable" was ticked in the database:

```
/* Define primary endpoint */
/* Causal Allergic Event */
DATA temsData; set temsData;
        if _011_ae_allerg_event=1 and
        (_015_ae_allerg_event_causali=2 or _015_ae_allerg_event_causali=3)
        then ae_ir_1=1;
        else if _011_ae_allerg_event=1 and
        (_015_ae_allerg_event_causali ne 2 and _015_ae_allerg_event_causali ne 3)
        then ae_ir_1=0;
        else if _011_ae_allerg_event=2
        then ae_ir_1=0;
        else ae_ir_1=.;
RUN;
/* Causal Intervention requiring RR decrease */
DATA temsData; set temsData;
        if _21_ae_bp_drop=1 and
        (_024_ae_bp_drop_causality=2 or _024_ae_bp_drop_causality=3)
        then ae_ir_2=1;
        else if _21_ae_bp_drop=1 and
        (_024_ae_bp_drop_causality ne 2 and _024_ae_bp_drop_causality ne 3)
        then ae_ir_2=0;
        else if _21_ae_bp_drop=2
        then ae_ir_2=0;
        else ae_ir_2=.;
RUN;
/* Causal Respiratory insufficiency during mission */
DATA temsData; set temsData;
        if _031_ae_resp_insuff=1 and
        (_034_ae_resp_insuff_causalit=2 or _034_ae_resp_insuff_causalit=3)
        then ae_ir_3=1;
        else if _031_ae_resp_insuff=1 and
        (_034_ae_resp_insuff_causalit ne 2 and _034_ae_resp_insuff_causalit ne 3)
        then ae_ir_3=0;
        else if _031_ae_resp_insuff=2
        then ae_ir_3=0;
        else ae_ir_3=.;
RUN;
/*Causal Cardiac arrest */
DATA temsData; set temsData;
        if _041_ae_cardiac_arrest=1 and
        (_044_ae_cardiac_arrest_causa=2  or _044_ae_cardiac_arrest_causa=3)
        then ae_ir_4=1;
        else if _041_ae_cardiac_arrest=1 and
        (_044_ae_cardiac_arrest_causa ne 2 and _044_ae_cardiac_arrest_causa ne 3)
        then ae_ir_4=0;
        else if _041_ae_cardiac_arrest=2
        then ae_ir_4=0;
        else ae_ir_4=.;
RUN;

/* At least one causal AE */
DATA temsData; set temsData;
        if ae_ir_1=1 or ae_ir_2=1 or ae_ir_3=1 or ae_ir_4=1
        then ae=1;
        else if ae_ir_1=0 and ae_ir_2=0 and ae_ir_3=0 and ae_ir_4=0
```

```
        then ae=0;
        else ae=.;
RUN;
```

1.2 DEFINITION OF SECONDARY ENDPOINTS AND OTHER OUTCOME MEASURES

1.2.1 OTHER INTERVENTION-RELATED ADVERSE EVENT

The secondary endpoint is defined by a status variable. The status variable is 1 if the patient experiences an intervention-related other AE and 0 if the patient experiences no other intervention related AE. The variables for the assessment of causality in the database have the 5 attributes “not confirmed”, “certain”, “probable”, “improbable”, “no relation”. The evaluation of the AEs of the clinical endpoint committee was judged by the CEC by the attributes “suspected” or “not suspected”. According to the initial version of the TSAP, an AE was only considered to have a sufficient causal relationship if "certain" was ticked in the database. As the attribute " suspected" was not available, we had to change the definition of an AE to be "suspected" of having a causal relationship if either the attribute "certain" or "probable" was ticked in the database:

```
/* Secondary endpoint: other intervention-related AE */
/* Causal other Adverse event */
DATA temsData; set temsData;
        if _51_ae_other=1 and (_055_ae_other_causality=2 or _055_ae_other_causality=3)
        then ae_other=1;
        else if _51_ae_other=1 and (_055_ae_other_causality ne 2 and _055_ae_other_causality
ne 3)
        then ae_other=0;
        else if _51_ae_other=2
        then ae_other=0;
        else ae_other=.;
RUN;
```

1.2.2 QUALITY OF DOCUMENTATION

The secondary endpoint quality of documentation (8.4.3) is defined by a score, which has to be changed because of variable coding in the database for the physician protocol and the paramedic protocol:

```
/* Secondary endpoint: Quality of documentation */
DATA temsData; set temsData;
        * Doctors protocol;
        if  _502_proto=1 then do;
                quality_documentation=((_1596a_naca_1 ne 2 or _1201_naca_N ne 2)+
                (_1340_NRS_1 ne .)+(_1350_NRS_N ne .)+
                (_1370a_ECG_rhythm_5_1 ne '2' and _1370a_ECG_rhythm_5_2 ne '2' and
                _1370a_ECG_rhythm_5_3 ne '2' and _1370a_ECG_rhythm_5_4 ne '2')+
                (_1360a_ECG_rhythm_4_1 ne '2' and _1360a_ECG_rhythm_4_2 ne '2' and
                _1360a_ECG_rhythm_4_3 ne '2' and _1360a_ECG_rhythm_4_4 ne '2')+
                (_1300_GCS_1 ne .)+(_1310_GCS_N ne .)+
                (_1260_RR_2 ne .)+(_1270_RR_N ne .)+(_1280_spO2_2 ne .)+
                (_1290_spO2_N ne .)+(_1240_HR_2 ne .)+(_1250_HR_N ne .)+
                (_1210_NIBP_sys_2 ne .)+(_1220_NIBP_sys_N ne .)+(_1215_NIBP_dia_2 ne .)+
                (_1225_NIBP_dia_N ne .)+(Sample=1)+(_1516_dia=1))/19; end;
        * Emergency protocol;
        else if _502_proto=2 then do;
                if _503_proto=1 then do;
                        quality_documentation=((_3318_NRS =1)+(_4318_NRS =1)+
                        (_3404_EKG=1 or _4001_measuresrettass_1 =16 or
                        _4001_measuresrettass_2 =16 or _4001_measuresrettass_3 =16 or
                        _4001_measuresrettass_4 =16 or _4001_measuresrettass_5 =16 or
                        _4001_measuresrettass_6 =16 or _4001_measuresrettass_7 =16 or
                        _4001_measuresrettass_8 =16)  +(_3314_AF=1)+ (_4314_AF=1)+
                        (_3312_SPO2=1)+ (_4312_SPO2=1)+ (_3310_HF=1)+
                        (_4310_HF=1)+(_3308_RR=1)+(_4308_RR=1)+(_3309_RR=1)+
                        (_4309_RR=1)+(Sample=1)+(_6108_dia=1))/15; end;
                else do; quality_documentation=.; end; end;
```

```
        else if _502_proto=. then do;
                if _503_proto=1 then do;
                        quality_documentation=((_3318_NRS =1)+(_4318_NRS =1)+
                        (_3404_EKG=1 or _4001_measuresrettass_1 =16 or
                        _4001_measuresrettass_2 =16 or _4001_measuresrettass_3 =16 or
                        _4001_measuresrettass_4 =16 or _4001_measuresrettass_5 =16 or
                        _4001_measuresrettass_6 =16 or _4001_measuresrettass_7 =16 or
                        _4001_measuresrettass_8 =16 ) +(_3314_AF=1)+ (_4314_AF=1)+
                        (_3312_SPO2=1)+ (_4312_SPO2=1)+(_3310_HF=1)+
                        (_4310_HF=1)+(_3308_RR=1)+(_4308_RR=1)+(_3309_RR=1)+
                        (_4309_RR=1)+(Sample=1)+(_6108_dia=1))/15; end;
                else do; quality_documentation=.; end; end;
        else do; quality_documentation=.; end;
RUN;
```

1.2.3 DURATION OF PHYSICIAN ENGAGEMENT-TIME

The secondary endpoint duration of physician engagement-time (8.4.4) is defined as a time variable that has to be changed to a missing in the case of no physicians contact:

```
/* Secondary endpoint: Duration of physician engagement time */
DATA temsData; set temsData;
        * Group NA;
        if      _1010_group=2 then do;
                if _1002_konv=2 then do;
                        Phys_engag_time = mod((_6605_time_operational_readi -
                        _6601_time_alarm1 + '24:0't),'24:0't); end;
                else if _1002_konv=1 then do;
                        Phys_engag_time = mod((_6013_TNA -
                        _6601_time_alarm1 + '24:0't),'24:0't); end;
                else do; Phys_engag_time=.; end; end;
        * Group TNA;
        else if _1010_group=1 then do;
                if _1002_konv=2 then do;
                        if _502_proto=1 then do;
                                Phys_engag_time = mod((_6013_TNA -
                                _6012_TNA + '24:0't),'24:0't); end;
                        else if _502_proto=2 then do;
                                Phys_engag_time=.; end;
                        else do; Phys_engag_time=.; end; end;
                else if _1002_konv=1 then do;
                        Phys_engag_time = mod((_6605_time_operational_readi -
                        min(_6012_TNA,_6601_time_alarm1) + '24:0't),'24:0't); end;
                else do; Phys_engag_time=.; end; end;
        else do; Phys_engag_time=.; end;
        format Phys_engag_time hhmm4.;
RUN;
```

1.2.4 TIME SPAN UNTIL HOSPITAL ARRIVAL

The outcome measure time span until arrival at hospital (8.5.1) is defined as a time variables that has to be changed to account for time points that occur on different dates:

```
/* Other outcome measure: time span until arrival at hospital */
DATA temsdata; set temsdata;
        if _1120_transport=1 then do;
                time_arrival=mod((_6009_RTW-_6006_RTW1 + '24:0't),'24:0't); end;
        else do time_arrival=.; end;
RUN;
```

1.3 MINOR CHANGES

Minor changes include the correction of typos and code errors:

- 8.4.7 REQUIRED CONVERSION FROM TELE-EMS TO CONVENTIONAL EMS PHYSICIAN:

```
/* Secondary endpoint: Required conversion from tele-EMS to conventional EMS physician */
DATA temsData; set temsData;
       if _1010_group=1 and _1002_konv=1
       then req_conversion=1;
       else if _1010_group=1 and _1002_konv ne 1
       then req_conversion=0;
       else req_conversion=.;
RUN;
```

- 8.5.2 CONVERSION OF EMS PHYSICIAN TO TELE-EMS PHYSICIAN

```
/*Conversion of EMS physician to tele-EMS physician*/
DATA temsData; set temsData;
       if _1010_group=2 and _1002_konv=1 then konv_EMS=1;
       else konv_EMS=0;
RUN;
```

2. SIGNATURES

Agreement to the Amendment of the Trial Statistical Analysis Plan:

Principal Investigator (gem §4 Abs. 25 AMG):

	Aachen, den 10.03.2022
Univ. Prof. Dr. med. Rolf Rossaint	Place / Date

Biostatistician:

	Aachen, 10.3.22
Univ. Prof. Dr. rer. nat. Ralf-Dieter Hilgers	Place / Date

TRIAL STATISTICAL ANALYSIS PLAN

TELEMEDICAL SUPPORT FOR PREHOSPITAL EMERGENCY MEDICAL SERVICE – A PROSPECTIVE RANDOMISED CONTROLLED TRIAL (TEMS-TRIAL):

VERSION 1.0, 07.07.2020

**Sponsor**	**RWTH Aachen**
**Clinical Trials.gov**	**NCT02617875**
**Principal Investigator (gem §4 Abs. 25 AMG)**	Univ. Prof. Dr. med. Rolf Rossaint<br>Department of Anaesthesiology<br>Medical Faculty, RWTH Aachen University<br>Pauwelsstr. 30,<br>52074 Aachen<br>Germany
**Biostatistician**	Univ. Prof. Dr. rer. nat. Ralf-Dieter Hilgers<br>Department of Medical Statistics<br>Medical Faculty, RWTH Aachen University<br>Pauwelsstr. 19,<br>52074 Aachen<br>Germany

TABLE OF CONTENTS

LIST OF ABBREVIATIONS 4
1. INTRODUCTION 5
2. SOFTWARE UTILIZED 5
2.1 OPERATING SYSTEM 5
2.2 SAMPLE SIZE CALCULATION 5
2.3 RANDOMISATION ALGORITHM 5
2.4 DATABASE 5
2.5 STATISTICAL ANALYSIS 5
3. STUDY DESIGN 5
4. STUDY OBJECTIVES 6
4.1 PRIMARY ENDPOINT 6
4.2 SECONDARY ENDPOINTS 7
4.3 OTHER OUTCOME MEASURES 7
4.4 MEASUREMENT METHODS OF ENDPOINTS 8
4.5 SAFETY PARAMETERS 8
5. STUDY SCHEDULE 9
6. POPULATION AND ASSOCIATED ANALYSIS 10
7. TARGET VARIABLES 10
7.1 BASELINE CHARACTERISTICS & OTHER OUTCOME MEASURES 10
7.2 VARIABLES EVALUATED FOR EFFICACY 12
7.2.1 PRIMARY ENDPOINT 12
7.2.2 SECONDARY ENDPOINTS 12
7.3 VARIABLES EVALUATED FOR SAFETY 13
7.4 VARIABLES EVALUATED FOR COMPLIANCE 14
8. DATA HANDLING 14
8.1 DATA VALIDATION 14
8.1.1 DATABASE 14
8.1.2 ENSURING DATA QUALITY 15
8.1.2.1 DATA ENTRY AND DOUBLE DATA RECONCILIATION 15
8.1.2.2 QUERY MANAGEMENT 15
8.1.3 DISCREPANCY MANAGEMENT 15
8.1.3.1 VERIFICATION FOR CORRECTNESS 15
8.1.3.2 PLAUSIBILITY CHECKS 15
8.2 DATE AND TIME VARIABLES 15
8.3 DEFINITION OF THE PRIMARY ENDPOINT 16
8.4 DEFINITION OF SECONDARY ENDPOINTS 16
8.4.1 QUALITY OF MEDICAL HISTORY SURVEY (SAMPLER) 16

8.4.2 TREATMENT QUALITY ... 17
8.4.3 QUALITY OF DOCUMENTATION ... 17
8.4.4 DURATION OF PHYSICIAN ENGAGEMENT-TIME ... 18
8.4.5 NON-INTERVENTION RELATED ADVERSE EVENTS ... 18
8.4.6 OTHER INTERVENTIION RELATED ADVERSE EVENTS ... 19
8.4.7 REQUIRED CONVERSION FROM TELE-EMS TO CONVENTIONAL EMS PHYSICIAN ... 20
8.4.8 ASSESSMENT AS TO WHETHER CONVENTIONAL EMS-PHYSICIAN OPERATION COULD HAVE BEEN HANDLED BY A TELE-EMS PHYSICIAN ... 20
8.4.9 DEATH WITHIN 24 HOURS ... 20
8.4.10 DEATH UNTIL HOSPITAL DISCHARGE ... 20
8.5 OTHER OUTCOME MEASURES ... 21
8.5.1 TIME SPAN UNTIL PHYSICIAN CONTACT AND HOSPITAL ARRIVAL ... 21
8.5.2 CONVERSION OF EMS PHYSICIAN TO TELE-EMS PHYSICIAN ... 21
8.6 UNBLINDING ... 21
8.7 DERANDOMISATION ... 21
9. DESCRIPTIVE STATISTICS ... 22
9.1 BASELINE CHARACTERISTICS & OTHER OUTCOME MEASURES ... 22
9.2 VARIABLES EVALUATED FOR EFFICACY ... 22
9.2.1 PRIMARY ENDPOINT ... 22
SECONDARY ENDPOINTS ... 23
9.2.1.1 DICHOTOMOUS AND CATEGORICAL VARIABLES ... 23
9.2.1.2 CONTINUOUS AND QUASI CONTINUOUS VARIABLES ... 24
9.3 VARIABLES EVALUATED FOR SAFETY ... 24
9.4 VARIABLES EVALUATED FOR COMPLIANCE ... 24
9.5 GRAPHICAL PRESENTATION ... 24
10. INFERENTIAL STATISTICS ... 25
10.1 PRIMARY ENDPOINT ... 25
10.2 SECONDARY ENDPOINTS ... 26
10.2.1 DICHOTOMOUS SECONDARY ENDPOINTS ... 26
10.2.2 CONTINUOUS AND QUASI CONTINUOUS SECONDARY ENDPOINTS ... 26
10.3 MULTIPLICITY ... 27
10.4 SAMPLE SIZE AND STATISTICL POWER ... 27
10.5 INTERIM ANALYSES ... 27
10.5.1 DECISION RULES OF INTERIM ANALYSES ... 28
11. FUTURE ANALYSES IN EXTENSION TO THE ACTUAL EVALUATION ... 28
12. PREPARATION AND QUALITY CONTROL OF PROGRAM CODE ... 28
12.1 STORAGE OF LOG FILES ... 28
12.2 CONTROL OF PROGRAM ... 28
13. CHANGES FROM STUDY PROTOCOL ... 29
14. REFERENCES ... 29
15. SIGNATURES ... 30

LIST OF ABBREVIATIONS

(S)AE	(Serious) Adverse Event
AHA	American Heart Association
CEC	Clinical endpoint committee
COPD	Chronic obstructive pulmonary disease
CRF	Case Report Form
CTC-A	Clinical Trial Center Aachen
DFG	German Research Foundation
DIN	German industrial standard
DIVI	German Interdisciplinary Society for Intensive and Emergency Medicine
DSMC	Data safety monitoring committee
ECG	electrocardiogram
e-CRF	Electronic case report form
e.g.	for example
EMS	Emergency Medical Service
h	hours
ICD 10	International Classification of Diseases
ICH-GCP	International Conference on Harmonisation of Technical Requirements for Registration of Pharmaceuticals for Human Use - Good Clinical Practice
ICU	Intensive Care Unit
IT	Information technology
NACA	National Advisory Committee for Aeronautics
RettG NRW	Law on Rescue Services of the state of North Rhine-Westphalia
SD	Standard Deviation
SOP	Standard Operating Procedures
STEMI	ST-elevation myocardial infarction
StGB	German Penal Code
tele-EMS physician	teleconsultation EMS physician

1. INTRODUCTION

The Trial Statistical Analysis Plan is a detailed technical extension to the clinical study protocol and follows the principles of the guidelines ICH E3, E6, E8, E9 and the Consort Statement. It has to be finished before the first interim analysis. It is related to:

Document	Version	Relation
Study Protocol	Version 6.0, 11.05.2017	Description of study design and objectives
E Case Report Forms:		Detailed description of all variables
Unerwünschte Ereignisse	Version 26	
Krankenhaus	Version 15	
Notarzt	Version 49	
Rett	Version 14	
Patientenfragebogen	Version 17	
End of Trial	Version 1	
Einwilligung	Version 20	

2. SOFTWARE UTILIZED

2.1 OPERATING SYSTEM

Current operating system is Windows 10 Professional. In the event of an update to a new version of the operating system, the new system is then used.

2.2 SAMPLE SIZE CALCULATION

Addplan 6.0 was applied.

2.3 RANDOMISATION ALGORITHM

According to technical conditions the randomisation procedure is implemented in the software of control center. The software is provided by the company ISE GmbH, Schönebergstr. 15, 52068 Aachen. Therefore, other than initially planned, ISE and the Department of Anaesthesiology are responsible for randomisation including assessment of appropriate procedure. It is no longer the responsibility of the Department of Medical Statistics (Amendment 2).

2.4 DATABASE

Initially Open Clinica, Version 3.12 was used, and in the course of updates Version 3.14 is currently being used.

2.5 STATISTICAL ANALYSIS

SAS (Version 9.4, SAS Institute Inc, Cary, NC, USA) is used for statistical analyses. In the event of an update to a new version of SAS, the new version is then used.

3. STUDY DESIGN

The TEMS-trial is a single-center, prospective, randomised, interventional, open label, two-arm parallel group sequential, non-inferiority trial. Two treatments will be compared in different groups providing a two-arm parallel group design. The study design is described in Figure 1.

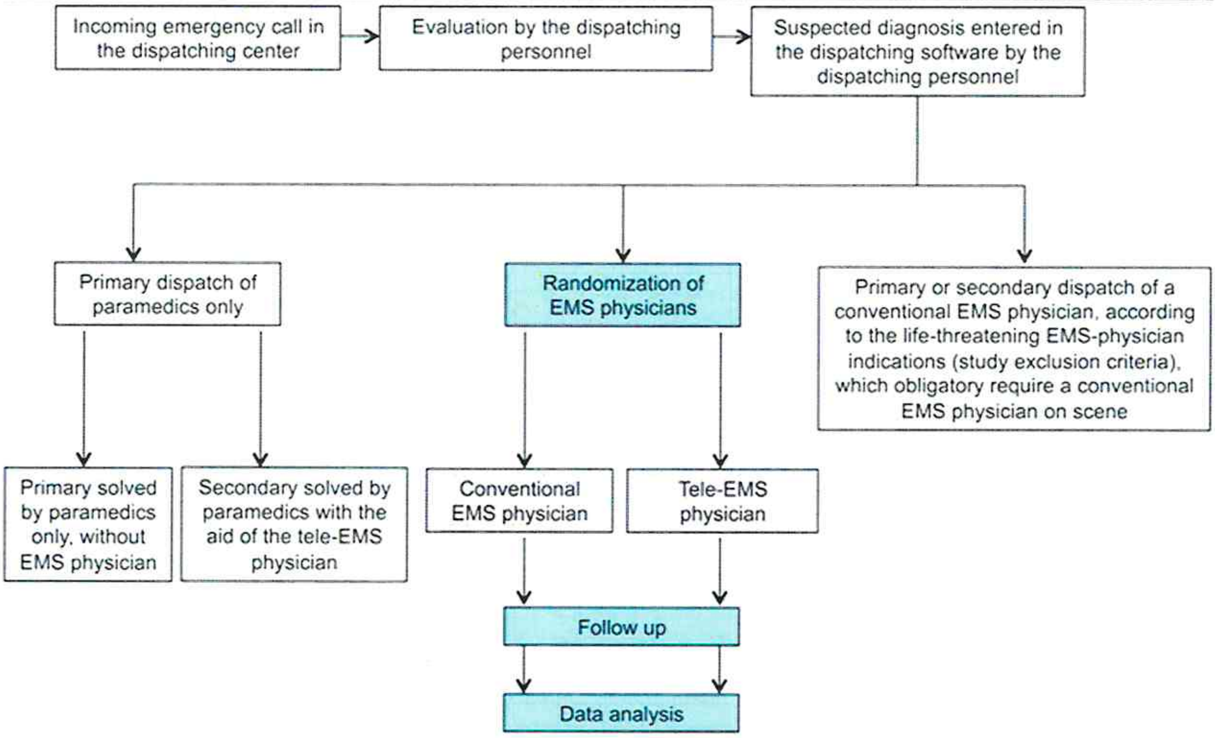

Figure 1: TEMS-trial study design flow chart

4. STUDY OBJECTIVES

Assess the quality of pre-hospital emergency care using the multifunctional teleconsultation system between paramedics and experienced tele-EMS physicians.

4.1 PRIMARY ENDPOINT

The specific primary objective is to determine if the primary usage of a tele-EMS physician compared to a conventional EMS physician on scene, in non-life-threatening cases, is non-inferior with regard to the occurrence of intervention-related patient AEs. The primary outcome measure will include the rate of AEs depending on the specific treatment by an EMS physician or a tele-EMS physician. A Clinical Endpoint Committee (CEC) will perform the endpoint adjudication by blinded evaluation and assignment of AEs into intervention-related AEs, according to predefined criteria.

These intervention-related AEs are defined as follows:

- Immediate allergic reaction to drug administration due to incorrect survey of patients' medical history (omission of the question for drug allergies).
- Intervention-related and immediate treatment-requiring hypotensive episode on scene (e.g., after wrong drug dosing or drug selection).
- Immediate intervention-related apnea or respiratory insufficiency on scene (e.g., after wrong drug dosing or drug selection).
- Intervention-related circulatory arrest within 24 h of EMS treatment (e.g., after wrong drug dosing, drug selection, or wrong referral to a hospital, which is not-specialized for the respective emergency case).

4.2 SECONDARY ENDPOINTS

The specific secondary objective is to determine if the primary usage of a tele-EMS physician compared to a conventional EMS physician on scene, in non-life-threatening cases, is superior in regard to the quality of the medical history survey, medical treatment and documentation, despite a shorter physician-engagement time. The secondary outcome measures will record the specific treatment-associated quality indicators depending on the use of an EMS physician or tele-EMS physician. These specific treatment-associated quality indicators are defined as:

- Quality of medical history survey (adherence to the guidelines).
- Treatment quality (adherence to the guidelines).
- Quality of documentation
- Duration of the physician engagement-time.
- Frequency of predefined "Tracer" diagnoses (with physician contact):
  - Trauma
  - Stroke
  - Acute coronary syndrome
  - Pain control
  - Bronchial asthma
  - Chronic obstructive pulmonary disease (COPD)
  - Seizure
  - Sepsis
  - Hypoglycemia
- AEs (independent of the kind of EMS care, e.g., allergic reaction despite adequate survey of medical history, nonintervention related hypotensive episode, apnea, cardiac arrest or other).
- Other intervention-related AEs
- Premature termination of the telemedical or conventional EMS operation due to it being unnecessary.
- Required conversion from a primarily dispatched tele-EMS physician to a conventional EMS physician.
- Assessment as to whether a conventional EMS physician operation could have been handled by a tele-EMS physician.
- Death within 24 h, after day 30 and until day 90 of hospitalization, respectively, until discharge from hospital.
- Intensive Care Unit (ICU) and hospital length of stay

4.3 OTHER OUTCOME MEASURES

The following variables will be assessed in each randomisation group:

- Time point of the first contact with a physician, time span between the emergency call and hospital arrival in each randomisation group
- Seven-step National Advisory Committee for Aeronautics (NACA) severity score in each randomisation group
- Proportion of conventional emergency cases, which were passed to a tele-EMS physician
- Assessment as to whether an EMS physician was even necessary for each emergency case
- Technical performance of the Tele-EMS physician
- Discharge destination from hospital in each randomisation group
- Frequency of tele-EMS contacting by a conventional EMS physician for any kind of advice

- Assessment of the medical education/experience of the involved physicians in each randomisation group

4.4 MEASUREMENT METHODS OF ENDPOINTS

The intervention-related AEs are counted to calculate the rate of AEs depending on the specific treatment.

4.5 SAFETY PARAMETERS

The intervention-related AEs are defined as follows:

- Immediate allergic reaction to drug application due to incorrect survey of patients` medical history (omission of the question for drug allergies).
- Intervention-related and immediate treatment-requiring blood pressure drop on scene (e.g. after wrong drug dosage or drug selection).
- Immediate intervention-related apnea or respiratory insufficiency on scene (e.g. after wrong drug dosage or drug selection).
- Intervention-related circulatory arrest within 24h of EMS treatment (e.g. after wrong drug dosage, drug selection, or wrong hospital referral).

The non-intervention-related AEs are defined as follows:

- AEs (independently of the kind of EMS care, e.g. allergic reaction despite adequate survey of medical history, not-intervention related blood pressure dropping, apnea or cardiac arrest)

The frequency of the intervention-related AEs as well as non-intervention-related AEs and resuscitation within 24h of hospital admission will be referred to the safety board committee.

Definition of AEs

All unexpected AEs and the expected primary outcome AEs in the course of the EMS treatment must be documented on an AE Reporting Form which will be provided by the sponsor. All investigators will receive intensive training by the sponsor regarding the definition, documentation and reporting of AEs. Each AE must be specified as follows:

1. Duration
2. Severity (mild, moderate, or severe)
3. Causal relationship to the treatment (suspected/not suspected)
4. Required treatment of AE and action taken with trial drug
5. Outcome
6. Seriousness

Definition of SAEs

AN SAE is an AE which:

1. Results in death (i.e., is fatal)
2. Is immediately life-threatening
3. Results in persistent or significant disability/incapacity
4. Requires or prolongs patient hospitalization
5. Results in a congenital anomaly/birth defect

6. May jeopardize the patient and may require medical or surgical intervention to prevent one of the aforementioned outcomes (for example, intensive treatment in an emergency room without hospitalization)

SAEs must be reported by the principal investigator to the sponsor and the DSMC within 24 h after detection. All SAEs will be summarized in the sponsor's Annual Safety Report.

5. STUDY SCHEDULE

A time schedule according to the SPIRIT figure is shown in Additional file 2.

Run-in phase

One or two months prior to the main study, we will assess the same outcomes as in the main study.

Phase A: enrollment

The dispatching personnel in the EMS dispatching center in Aachen will screen all emergency calls for eligibility and enter a suspected diagnosis into the dispatching software.

Phase B: allocation

All non-life-threatening emergency calls, which do not obligatory require an EMS physician on scene but cannot solely be resolved by the paramedics, will be randomised into the two intervention groups (conventional EMS physician and tele-EMS physician, respectively) automatically by the dispatching software.

Phase C: during EMS intervention, post allocation

Patients will be treated by both kinds of physicians according to the SOP and all operation-related data will be documented in a standardized EMS Documentation Form according to the recommendation of the German Interdisciplinary Society for Intensive and Emergency Medicine (DIVI). As usual in the present routine, the patients in the tele-EMS-physician group will be verbally informed about the use of a tele-EMS physician and the teleconsultation system. Written informed consent for study participation will be obtained as soon as possible during this phase until discharge from hospital.

Phase D: early follow-up, post-allocation

A survey of the patients regarding their satisfaction with the EMS system used will be conducted after hospital arrival until discharge, if the patients have written consented to these further assessments. Assessment of the outcome death within 24 h and until hospital discharge, respectively. Assessment of ICU and hospital length of stay and the discharge diagnosis of the hospital.

Phase E: late follow-up, close-out maximum until day 90

Additionally, we will assess the mortality within 30 and 90 days, respectively.

Phase F: additional analysis, parallel to the main study

Retrospective analysis will be made of the nonrandomised, with conventional EMS-Physician-treated patients. A satisfaction survey of the paramedics, EMS physicians and emergency room personnel will also be undertaken.

6. POPULATION AND ASSOCIATED ANALYSIS

According to the [ICH Topic E 9] there are two different sets (full analysis set and per protocol set) for the statistical analysis. Both sets are described with respect to the study in the following sections.

Full Analysis Set (Intention-to-Treat Sample)

The term 'full analysis set' describes the analysis set which is as complete as possible and as close as possible to the intention-to-treat ideal of including all randomised subjects.

The following circumstances lead to excluding randomised subjects from the full analysis set:

- failure to satisfy major entry criteria (eligibility violations);
- lack of any data post randomisation.

Subjects who fail to satisfy an entry criterion may be excluded without introducing bias only if:

- the entry criterion was measured prior to randomisation;
- the detection of relevant eligibility violations can be made completely objective;
- all subjects receive equal scrutiny for eligibility violations;
- all detected violations of the particular entry criterion are excluded.
- Potential biases arising from these specific exclusions, or any others, must be addressed.

Differences between randomised subjects and full analysis set will be reported.

Per Protocol Set

Per protocol set is defined as subset of the full analysis set. First, patients who are not treated per protocol are excluded. Second, subjects are excluded, of whom not all information for the primary endpoint are available.

The hybrid analysis population as developed by Sanchez will be used as analysis population for the primary endpoint (Sanchez 2006).

The full analysis set is used to analyse secondary endpoints.

7. TARGET VARIABLES

7.1 BASELINE CHARACTERISTICS & OTHER OUTCOME MEASURES

In Table 1 all baseline characteristics as well as other outcome measures with unit and data type are given. The exact definition of some of these outcome measures is explained in section 8.5.

Variable	Description	Unit	Data type
_1230_age	Age	Years	Continuous
_1140_sex_4593	Sex		Dichotomous
time_physician	Time span until first physician contact	hh:mm	Continuous
time_arrival	Time span until arrival at hospital	hh:mm	Continuous
_1596a_naca_1__4869	NACA classification first diagnosis		Categorical
1201_naca_N_4149	NACA classification transfer		Categorical

konv_EMS	Proportion of conventional emergency cases, which were passed to a tele-EMS physician		Dichotomous
_2061_conv_EP_necessity	Necessity of EMS physician		Categorical
_6016_TNA	Necessity of Tele-EMS physician		Categorical
technical_performace	Technical performance		Dichotomous
_8003_outcome_discharge_des_6130	Discharge destination from hospital		Categorical
medical_education	Medical education/experience of involved physicians		Categorical
md_acs	Frequency of main diagnoses for all emergency cases: Acute coronary syndrome		Dichotomous
md_rhythm	Frequency of main diagnoses for all emergency cases: Rhythm disorder		Dichotomous
md_cardiovascular	Frequency of main diagnoses for all emergency cases: Cardiovascular disorder		Dichotomous
md_pulmonary	Frequency of main diagnoses for all emergency cases: Pulmonary disorder		Dichotomous
md_neurology	Frequency of main diagnoses for all emergency cases: Neurologic disorder		Dichotomous
md_intox	Frequency of main diagnoses for all emergency cases: Intoxication		Dichotomous
md_psych	Frequency of main diagnoses for all emergency cases: Psychiatric disorder		Dichotomous
md_general_condition	Frequency of main diagnoses for all emergency cases: Reduced general condition		Dichotomous
md_metabolism	Frequency of main diagnoses for all emergency cases: Metabolic disorder		Dichotomous
md_acute_abdomen	Frequency of main diagnoses for all emergency cases: Acute abdomen		Dichotomous
md_anaphylaxis	Frequency of main diagnoses for all emergency cases: Anaphylaxis		Dichotomous
md_abdominal disorder	Frequency of main diagnoses for all emergency cases: Abdominal disorder		Dichotomous
md_orthopaedic	Frequency of main diagnoses for all emergency cases: Orthopaedic		Dichotomous
md_trauma	Frequency of main diagnoses for all emergency cases: Trauma		Dichotomous

md_infection	Frequency of main diagnoses for all emergency cases: suspected infection		Dichotomous
md_other	Frequency of main diagnoses for all emergency cases: Other		Dichotomous

Table 1: Baseline characteristics & other outcome measures

7.2 VARIABLES EVALUATED FOR EFFICACY

7.2.1 PRIMARY ENDPOINT

A Clinical Endpoint Committee (CEC) will perform the endpoint adjudication by blinded evaluation and assignment of AEs into intervention-related AEs, according to predefined criteria.

All adverse events will be evaluated by the blinded CEC, who assess which adverse events of the four pre-defined categories (see 4.1) are induced by the randomly allocated kind of treatment (tele-EMS physician or conventional physician, respectively). As result, we get the rate of AEs depending on the specific treatment by an EMS physician or a tele-EMS physician. The variable regarding the primary endpoint is given in Table 2. The exact definition of the primary endpoint is explained in section 8.3.

**Variable**	**Description**	**Data type**
ae	At least one AE induced by treatment	Dichotomous

Table 2: Primary endpoint

7.2.2 SECONDARY ENDPOINTS

All variables regarding the secondary endpoints are given in Table 3. The exact definition of some of the secondary endpoint variables is explained in section 8.4.

**Variable**	**Description**	**Data type**
Sample	Quality of medical history survey (SAMPLE)	Continuous
Sample_r	Quality of medical history survey regarding the “R”=risk factor)	Dichotomous
Hospital_choice_correct	Treatment quality: Choice of the correct hospital	Dichotomous
ECG_used	Treatment quality: was an electrocardiogram applied to the patient?	Categorical
NIBP_measured	Treatment quality: was the patient’s blood pressure measured non-invasively?	Dichotomous
spO2_measured	Treatment quality: was the patient’s oxygen saturation measured?	Dichotomous
Glucose_measured	Treatment quality: was the patient’s blood glucose measured?	Dichotomous
iv_cannula_placed	Treatment quality: was an intravenous access successfully established?	Dichotomous
Quality_documentation	Quality of documentation	Continuous
Phys_engag_time	Duration of physician engagement-time	Continuous
8000_INJURY_9609	Frequency of “Tracer” diagnosis (with physician contact): Trauma	Dichotomous
_5200_stroke	Frequency of “Tracer” diagnosis (with physician contact): Stroke	Dichotomous
_5200_ACS1	Frequency of “Tracer” diagnosis (with physician contact): Acute coronary syndrome	Dichotomous

_5330_pain1	Frequency of "Tracer" diagnosis (with physician contact): Pain control	Dichotomous
_5550_asthma	Frequency of "Tracer" diagnosis (with physician contact): bronchial asthma	Dichotomous
_5660_copd1	Frequency of "Tracer" diagnosis (with physician contact): Chronic obstructive pulmonary disease (COPD)	Dichotomous
_5770_seizure	Frequency of "Tracer" diagnosis (with physician contact): Seizure	Dichotomous
_5800_sepsis_313	Frequency of "Tracer" diagnosis (with physician contact): Sepsis	Dichotomous
_5440_hypoglyc	Frequency of "Tracer" diagnosis (with physician contact): Hypoglycemia	Dichotomous
ae_nir	Non-Intervention-related Adverse Event	Dichotomous
ae_nir_1	Non-Intervention-related allergic event	Dichotomous
ae_nir_2	Non-Intervention-related intervention requiring RR decrease	Dichotomous
ae_nir_3	Non-Intervention-related respiratory insuffiency during mission	Dichotomous
ae_nir_4	Non-Intervention-related cardic arrest	Dichotomous
ae_nir_5	Non-Intervention-related other Adverse Event	Dichotomous
ae_other	Intervention-related other Adverse Event	Dichotomous
premature_termination	Premature termination of the telemedical or conventional EMS operation due to it being unnecessary	Dichotomous
Req_conversion	Required conversion from tele-EMS to conventional EMS-physician	Dichotomous
handled_by_tele_ems	Assessment as to whether conventional EMS-physician operation could have been handled by a tele-EMS physician	Dichotomous
Death_24_h	Death within 24 hours	Dichotomous
_8004_outcome_30d_fu_7268	Death within 30 days	Dichotomous
_8005_outcome_90d_fu_2853	Death within 90 days	Dichotomous
Death_discharge	Death until hospital discharge	Dichotomous
_8002_outcome_hospital_leng_8648	Hospital length of stay	Continuous
_8001_outcome_icu_length_of_9421	Intensive care unit length of stay	Continuous

Table 3: Secondary endpoints

7.3 VARIABLES EVALUATED FOR SAFETY

In Table 4 all adverse events with unit and data type are given.

Variable	Description	Data type
_011_ae_allerg_event	Allergic Event	Dichotomous
_13_ae_allerg_event_duration	Duration of allergic event	Dichotomous
_14_ae_allerg_event_intensit	Intensity of allergic event	Categorical
_015_ae_allerg_event_causali	Causality of allergic event	Categorical
_16_ae_allerg_event_treatmen	Treatment of the allergic event	Categorical
_017_ae_allerg_event_outcome	Allergic Event Outcome	Categorical

_18_ae_allerg_event_sae	Serious Allergic Event	Dichotomous
_21_ae_bp_drop	Intervention requiring RR decrease	Dichotomous
_22_ae_bp_drop_duration	Duration of the BP drop	Dichotomous
_23_ae_bp_drop_intensity	Intensity of the BP drop	Categorical
_024_ae_bp_drop_causality	Causality of the BP drop	Categorical
_25_ae_bp_drop_treatment	Treatment of the BP drop	Categorical
_026_ae_bp_drop_outocme	Outcome of the BP drop	Categorical
_27_ae_bp_drop_sae	Serious BP drop event	Dichotomous
_031_ae_resp_insuff	Respiratory insufficiency during mission	Dichotomous
_32_ae_resp_insuff_duration	Duration of the respiratory insufficiency	Dichotomous
_33_ae_resp_insuff_intensity	Intensity of the respiratory insufficiency	Categorical
_034_ae_resp_insuff_causalit	Causality of the respiratory insufficiency	Categorical
_35_ae_resp_insuff_treatment	Treatment of the respiratory insufficiency	Categorical
_036_ae_resp_insuff_outocme	Outcome of the respiratory insufficiency	Categorical
_37_ae_resp_insuff_sae	Serious respiratory insufficiency event	Dichotomous
_041_ae_resp_insuff	Cardiac arrest	Dichotomous
_42_ae_cardiac_arrest_durati	Duration of cardiac arrest	Dichotomous
_43_ae_cardiac_arrest_intens	Intensity of cardiac arrest	Categorical
_044_ae_cardiac_arrest_causa	Causality of cardiac arrest	Categorical
_45_ae_cardiac_arrest_treatm	Treatment of the cardiac arrest	Categorical
_046_ae_cardiac_arrest_outoc	Outcome of cardiac arrest	Categorical
_47_ae_cardiacarrest_sae	Serious cardiac arrest event	Dichotomous
_51_ae_other	Other adverse event	Dichotomous
_052_ae_other_text	Description of other adverse event	Text
_53_ae_other_duration	Duration of other adverse event	Dichotomous
_54_ae_other_intensity	Intensity of other adverse event	Categorical
_055_ae_other_causality	Causality of other adverse event	Categorical
_56_ae_other_treatment	Treatment of the other adverse event	Categorical
_057_ae_other_outocme	Outcome of other adverse event	Categorical
_58_ae_other_sae	Serious other adverse event	Dichotomous

Table 4: Adverse events for safety

7.4 VARIABLES EVALUATED FOR COMPLIANCE

Not applicable.

8. DATA HANDLING

After closing of the OpenClinica database, the database will be exported in SAS format and saved in the following folder: V:\KlinischeStudien\TEMS\Daten\Analysedatenbank.

8.1 DATA VALIDATION

8.1.1 DATABASE

A table is created, in which all data that was previously available in different tables is merged. The data is available in one row for each patient.

8.1.2 ENSURING DATA QUALITY

8.1.2.1 DATA ENTRY AND DOUBLE DATA RECONCILIATION

Data entry into the ECRF will be performed according to the CRF Completion Guidelines prepared along with the data management plan. The user will receive a unique user ID with access limitation to data entry or quality check. This ensures that each user can access only the respective functionalities allotted to that user ID and cannot make any other change in the database. For responsibilities where changes are permitted to be made in the data, the software will record the change made, the user ID that made the change and the time and date of change, for audit purposes (audit trail).
To minimise input errors, after the first entry, another independent person will check the entered patient data for each patient. This will enable data verification and reconciliation by identification of transcription errors and discrepancies caused by illegible data.

8.1.2.2 QUERY MANAGEMENT

The Clinical Research Associate (CRA) of the Centre for Translational and Clinical Research (CTC-A) of the University Hospital RWTH Aachen will monitor the entries made in the ECRF for completeness and correctness for 10% of the included patients. The monitoring will be conducted according to the monitoring manual. The CRA will also manually check the paper-based source data for missing pages and illegible data, to assure that the data are not lost. In case of missing or illegible data, a clarification is obtained from the investigator and the issue is resolved.
Discrepancies are flagged to the investigator for clarification. The software will generate an online data clarification form (DCF) for the investigator. Investigators will provide a resolution or explain the circumstances that led to the discrepancy in data. Thereafter, this resolution will be updated in the database automatically. The CRA will review the proposed resolution and close the query.

8.1.3 DISCREPANCY MANAGEMENT

Validity of data will be checked in accordance with the protocol specifications. Edit check programs are written to identify the discrepancies in the entered data, which are embedded in the database, to ensure data validity. These programs are written according to the logic condition and tested with dummy data before inception of the trial. If a data entry fails to pass the validation check, a discrepancy note will be generated. The investigator will have to resolve the discrepancy note, in order to be able to store the entered data for the respective visit. All discrepancies will be recorded and stored with audit trail.

8.1.3.1 VERIFICATION FOR CORRECTNESS

The verification for correctness is the comparison of the entries within a variable with the data type defined for the variable. For example, a number variable must not contain letters.

Saving the data within the database is not possible unless this verification is successful.

8.1.3.2 PLAUSIBILITY CHECKS

The plausibility checks check whether the entries can occur in the individual variables (for example, value range checks). Additional plausibility checks are performed to verify the correctness of entries within a variable with regard to the interactions between individual variables.

8.2 DATE AND TIME VARIABLES

Date Variables are transformed to a SAS defined with SAS Format DATE9. Time variables are transformed to a SAS defined Variable with SAS Format Time5.

8.3 DEFINITION OF THE PRIMARY ENDPOINT

The primary endpoint is defined by a status variable. The status variable is 1 if the patient experiences at least on intervention-related AE and 0 if the patient experiences no intervention related AE.

```
/* Define primary endpoint */
/* Causal Allergic Event */
DATA temsData; set temsData;
        if _011_ae_allerg_event=1 and _015_ae_allerg_event_causali=2
        then ae_ir_1=1;
        else if _011_ae_allerg_event=1 and _015_ae_allerg_event_causali ne 2
        then ae_ir_1=0;
        else if _011_ae_allerg_event=2
        then ae_ir_1=0;
        else ae_ir_1=.;
RUN;
/* Causal Intervention requiring RR decrease */
DATA temsData; set temsData;
        if _21_ae_bp_drop=1 and _024_ae_bp_drop_causality=2
        then ae_ir_2=1;
        else if _21_ae_bp_drop=1 and _024_ae_bp_drop_causality ne 2
        then ae_ir_2=0;
        else if _21_ae_bp_drop=2
        then ae_ir_2=0;
        else ae_ir_2=.;
RUN;
/* Causal Respiratory insufficiency during mission */
DATA temsData; set temsData;
        if _031_ae_resp_insuff=1 and _034_ae_resp_insuff_causalit=2
        then ae_ir_3=1;
        else if _031_ae_resp_insuff=1 and _034_ae_resp_insuff_causalit ne 2
        then ae_ir_3=0;
        else if _031_ae_resp_insuff=2
        then ae_ir_3=0;
        else ae_ir_3=.;
RUN;
/* Causal Cardiac arrest */
DATA temsData; set temsData;
        if _041_ae_resp_insuff=1 and _044_ae_cardiac_arrest_causa=2
        then ae_ir_4=1;
        else if _041_ae_resp_insuff=1 and _044_ae_cardiac_arrest_causa ne 2
        then ae_ir_4=0;
        else if _041_ae_resp_insuff=2
        then ae_ir_4=0;
        else ae_ir_4=.;
RUN;
/* At least one causal AE */
DATA temsData; set temsData;
        if ae_ir_1=1 or ae_ir_2=1 or ae_ir_3=1 or ae_ir_4=1
        then ae=1;
        else if ae_ir_1=0 and ae_ir_2=0 and ae_ir_3=0 and ae_ir_4=0
        then ae=0;
        else ae=.;
RUN;
```

8.4 DEFINITION OF SECONDARY ENDPOINTS

8.4.1 QUALITY OF MEDICAL HISTORY SURVEY (SAMPLER)

The secondary endpoint quality of medical history survey is defined by a score that is calculated by:

```
/* Secondary endpoint: Quality of medical history survey (SAMPLER) */
DATA temsData; set temsData;
        * Doctors protocol;
        if      _502_proto=1 then do;
                Sample=((_3010_symptoms_7760=1)+(_3020_allergies_7866=1)+
                (_3030_medication_3762=1)+(_3040_past_medical_history_7594=1)+
                (_3050_last_oral_intake_709=1)+(_3060_events_prior_to_incide_771=1))/6;
                Sample_R=_3070_risk_factors_804; end;
        * Emergency protocol;
        else if _502_proto=2 then do;
                if _503_proto=1 then do;
```

```
                    Sample=((_6001_symptoms_9097=1)+(_6002_allergies_2066=1)+
                    (_6003_medication_3772=1)+(_6004_past_medical_history_9971=1)+
                    (_6005_last_oral_intake_4902=1)+
                    (_6006_events_prior_to_incide_250=1))/6;
                    Sample_R=_6007_risk_factors_6910; end;
            else do; Sample=.; Sample_R=.; end; end;
        else if _502_proto=. then do;
            if _503_proto=1 then do;
                    Sample=((_6001_symptoms_9097=1)+ (_6002_allergies_2066=1)+
                    (_6003_medication_3772=1)+ (_6004_past_medical_history_9971=1)+
                    (_6005_last_oral_intake_4902=1)+
                    (_6006_events_prior_to_incide_250=1))/6;
                    Sample_R=_6007_risk_factors_6910; end;
            else do; Sample=.; Sample_R=.; end; end;
        else do; Sample=.; Sample_R=.; end;
RUN;
```

8.4.2 TREATMENT QUALITY

The secondary endpoint treatment quality is defined as several items that are calculated by:

```
/* Secondary endpoint: Treatment quality */
DATA temsData; set temsData;
        * Doctors protocol;
        if      _502_proto=1 then do;
                hospital_choice_correct=(_4010_hospital_choice_corre_4674=1);
                ECG_used=(_4120_ECG_used=1);
                NIBP_measured=(_4130_NIBP_measured=1);
                spO2_measured=(_4041_spO2_measured=1);
                glucose_measured=(_4150_glucose_measured=1);
                iv_cannula_placed=(_4260_iv_cannula_placed=1); end;
        * Emergency protocol;
        else if _502_proto=2 then do;
                if _503_proto=1 then do;
                        hospital_choice_correct=(hosp_choice_amb_only=1);
                        ECG_used=(_3404_ekg=1 or _4001_measuresrettass_3384='16');
                        NIBP_measured=((_3308_RR=1 and _3309_RR=1)
                                or (_4308_RR=1 and _4309_RR=1));
                        spO2_measured=(_3312_SPO2=1 or _4312_SPO2=1);
                        glucose_measured=(_3316_BZ=1 or _4316_BZ=1);
                        iv_cannula_placed=(_4001_measuresrettass_3384='15'
                                or _4001_measuresrettass_3384='17'); end;
                else do; hospital_choice_correct=.; ECG_used=.; NIBP_measured=.;
                        spO2_measured=.; glucose_measured=.; iv_cannula_placed=.; end; end;
        else if _502_proto=. then do;
                if _503_proto=1 then do;
                        hospital_choice_correct=(hosp_choice_amb_only=1);
                        ECG_used=(_3404_ekg=1 or _4001_measuresrettass_3384='16');
                        NIBP_measured=((_3308_RR=1 and _3309_RR=1)
                                or (_4308_RR=1 and _4309_RR=1));
                        spO2_measured=(_3312_SPO2=1 or _4312_SPO2=1);
                        glucose_measured=(_3316_BZ=1 or _4316_BZ=1);
                        iv_cannula_placed=(_4001_measuresrettass_3384='15'
                                or _4001_measuresrettass_3384='17'); end;
                else do; hospital_choice_correct=.; ECG_used=.; NIBP_measured=.;
                        spO2_measured=.; glucose_measured=.; iv_cannula_placed=.; end; end;
        else do hospital_choice_correct=.; ECG_used=.; NIBP_measured=.;
                spO2_measured=.; glucose_measured=.; iv_cannula_placed=.; end;
RUN;
```

8.4.3 QUALITY OF DOCUMENTATION

The secondary endpoint quality of documentation is defined by a score that is calculated by:

```
/* Secondary endpoint: Quality of documentation */
DATA temsData; set temsData;
        * Doctors protocol;
        if      _502_proto=1 then do;
                quality_documentation=((_1340_NRS_1_849 ne .)+(_1350_NRS_N_921 ne .)+
                (_1360a_ECG_rhythm_4 ne '2')+(_1260_RR_2 ne .)+(_1270_RR_N_4834 ne .)+
                (_1280_spO2_2 ne .)+(_1290_spO2_N_6556 ne .)+(_1240_HR_2 ne .)+
```

```
                (_1250_HR_N_3526 ne .)+(_1210_NIBP_sys_2 ne .)+
                (_1220_NIBP_sys_N_6855 ne .)+(_1215_NIBP_dia_2 ne .)+
                (_1225_NIBP_dia_N_426 ne .)+(Sample=1)+(_1516_dia=1))/15; end;
        * Emergency protocol;
        else if _502_proto=2 then do;
                if _503_proto=1 then do;
                        quality_documentation=((_3318_NRS =1)+(_4318_NRS =1)+
                        (_3404_EKG=1 or _4001_measuresrettass_3384='16')+(_3314_AF=1)+
                        (_4314_AF=1)+(_3312_SPO2=1)+(_4312_SPO2=1)+(_3310_HF=1)+
                        (_4310_HF=1)+(_3308_RR=1)+(_4308_RR=1)+(_3309_RR=1)+
                        (_4309_RR=1)+(Sample=1)+(_6108_dia=1))/15; end;
                else do; quality_documentation=.; end; end;
        else if _502_proto=. then do;
                if _503_proto=1 then do;
                        quality_documentation=((_3318_NRS =1)+(_4318_NRS =1)+
                        (_3404_EKG=1 or _4001_measuresrettass_3384='16')+(_3314_AF=1)+
                        (_4314_AF=1)+(_3312_SPO2=1)+(_4312_SPO2=1)+(_3310_HF=1)+
                        (_4310_HF=1)+(_3308_RR=1)+(_4308_RR=1)+(_3309_RR=1)+
                        (_4309_RR=1)+(Sample=1)+(_6108_dia=1))/15; end;
                else do; quality_documentation=.; end; end;
        else do; quality_documentation=.; end;
RUN;
```

8.4.4 DURATION OF PHYSICIAN ENGAGEMENT-TIME

The secondary endpoint duration of physician engagement-time is defined as a time variable that is calculated by:

```
/* Secondary endpoint: Duration of physician engagement time */
DATA temsData; set temsData;
        * Group NA;
        if      _1010_group=2 then do;
                if _1002_konv=2 then do;
                        Phys_engag_time = mod((_6605_time_operational_readi -
                        _6601_time_alarm1 + '24:0't),'24:0't); end;
                else if _1002_konv=1 then do;
                        Phys_engag_time = mod((_6013_TNA -
                        _6601_time_alarm1 + '24:0't),'24:0't); end;
                else do; Phys_engag_time=.; end; end;
        * Group TNA;
        else if _1010_group=1 then do;
                if _1002_konv=2 then do;
                        if _502_proto=1 then do;
                                Phys_engag_time = mod((_6013_TNA -
                                _6012_TNA + '24:0't),'24:0't); end;
                        else if _502_proto=2 then do;
                                Phys_engag_time=0; end;
                        else do; Phys_engag_time=.; end; end;
                else if _1002_konv=1 then do;
                        Phys_engag_time = mod((_6605_time_operational_readi -
                        min(_6012_TNA,_6601_time_alarm1) + '24:0't),'24:0't); end;
                else do; Phys_engag_time=.; end; end;
        else do; Phys_engag_time=.; end;
        format Phys_engag_time hhmm4.;
RUN;
```

8.4.5 NON-INTERVENTION RELATED ADVERSE EVENTS

The secondary endpoint if a patient has at least one non intervention-related AE is a status variable that is calculated by:

```
/* Secondary endpoint: non intervention-related AEs */
/* Non-Causal Allergic Event */
DATA temsData; set temsData;
        if _011_ae_allerg_event=1 and _015_ae_allerg_event_causali=5
        then ae_nir_1=1;
        else if _011_ae_allerg_event=1 and _015_ae_allerg_event_causali ne 5
        then ae_nir_1=0;
        else if _011_ae_allerg_event=2
        then ae_nir_1=0;
```

```
        else ae_nir_1=.;
RUN;
/* Non-Causal Intervention requiring RR decrease */
DATA temsData; set temsData;
        if _21_ae_bp_drop=1 and _024_ae_bp_drop_causality=5
        then ae_nir_2=1;
        else if _21_ae_bp_drop=1 and _024_ae_bp_drop_causality ne 5
        then ae_nir_2=0;
        else if _21_ae_bp_drop=2
        then ae_nir_2=0;
        else ae_nir_2=.;
RUN;
/* Non-Causal Respiratory insufficiency during mission */
DATA temsData; set temsData;
        if _031_ae_resp_insuff=1 and _034_ae_resp_insuff_causalit=5
        then ae_nir_3=1;
        else if _031_ae_resp_insuff=1 and _034_ae_resp_insuff_causalit ne 5
        then ae_nir_3=0;
        else if _031_ae_resp_insuff=2
        then ae_nir_3=0;
        else ae_nir_3=.;
RUN;
/* Non-Causal Cardiac arrest */
DATA temsData; set temsData;
        if _041_ae_resp_insuff=1 and _044_ae_cardiac_arrest_causa=5
        then ae_nir_4=1;
        else if _041_ae_resp_insuff=1 and _044_ae_cardiac_arrest_causa ne 5
        then ae_nir_4=0;
        else if _041_ae_resp_insuff=2
        then ae_nir_4=0;
        else ae_nir_4=.;
RUN;
/* Non-Causal other Adverse event */
DATA temsData; set temsData;
        if _51_ae_other=1 and _055_ae_other_causality=5
        then ae_nir_5=1;
        else if _51_ae_other=1 and _055_ae_other_causality ne 5
        then ae_nir_5=0;
        else if _51_ae_other=2
        then ae_nir_5=0;
        else ae_nir_5=.;
RUN;
/* At least one non-causal AE */
DATA temsData; set temsData;
        if ae_nir_1=1 or ae_nir_2=1 or ae_nir_3=1 or ae_nir_4=1 or ae_nir_5=1
        then ae_nir=1;
        else if ae_nir_1=0 and ae_nir_2=0 and ae_nir_3=0 and ae_nir_4=0 and ae_nir_5=0
        then ae_nir=0;
        else ae_nir=.;
RUN;
```

8.4.6 OTHER INTERVENTIION RELATED ADVERSE EVENTS

The secondary endpoint if a patient has another intervention-related AE as defined for primary endpoint is a status variable that is calculated by:

```
/* Secondary endpoint: other intervention-related AE */
/* Causal other Adverse event */
DATA temsData; set temsData;
        if _51_ae_other=1 and _055_ae_other_causality=2
        then ar_other=1;
        else if _51_ae_other=1 and _055_ae_other_causality ne 2
        then ae_other=0;
        else if _51_ae_other=2
        then ae_other=0;
        else ae_other=.;
RUN;
```

8.4.7 REQUIRED CONVERSION FROM TELE-EMS TO CONVENTIONAL EMS PHYSICIAN

The secondary endpoint of a required conversion from tele-EMS to conventional EMS physician is a status variable that is calculated by:

```
/* Secondary endpoint: Required conversion from tele-EMS to conventional EMS physician */
DATA temsData; set temsData;
        if _1010_group=1 and _1002_konv=1
        then req_conversion=1;
        else if _1010_group=1 and _1002_konv ne 1
        then req_conversion=0;
        else req_coversion=.;
RUN;
```

8.4.8 ASSESSMENT AS TO WHETHER CONVENTIONAL EMS-PHYSICIAN OPERATION COULD HAVE BEEN HANDLED BY A TELE-EMS PHYSICIAN

The secondary endpoint assessment as to whether a conventional EMS-physician operation could have been handled by a tele-Ems physician is a status variable that is calculated by:

```
/* Secondary endpoint: Assessment as to whether conventional EMS-physician operation
could have been handled by a tele-EMS physician */
DATA temsData; set temsData;
        if _1010_group=2 and _2061_conv_EP_necessity=11
        then handled_by_tele_ems=1;
        else if _1010_group=2 and _2061_conv_EP_necessity ne 11
        then handled_by_tele_ems=0;
        else handled_by_tele_ems=.;
RUN;
```

8.4.9 DEATH WITHIN 24 HOURS

The secondary endpoint death within 24 hours is a status variable that is calculated by:

```
/* Secondary endpoint: Death within 24 hours */
DATA temsData; set temsData;
        if      (_8002_outcome_hospital_leng_8648<2 and
                _8003_outcome_discharge_des_6130=4) or
                (_041_ae_resp_insuff=1 and _1120_transport=2)
        then death_24_h=1;
        else if _8002_outcome_hospital_leng_8648>1 or
                _8004_outcome_30D_FU_7268=1
        then death_24_h=0;
        else death_24_h=.;
RUN;
```

8.4.10 DEATH UNTIL HOSPITAL DISCHARGE

The secondary endpoint death until hospital discharge is a status variable that is calculated by:

```
/* Secondary endpoint: Death until hospital discharge */
DATA temsData; set temsData;
        if _1120_transport=1 and _8003_outcome_discharge_des_6130=4
        then death_discharge=1;
        else if _8003_outcome_discharge_des_6130 ne 4 and
                _8003_outcome_discharge_des_6130 ne .
        then death_discharge=0;
        else if _1120_transport=1 and _8003_outcome_discharge_des_6130=.
        then death_discharge=.;
RUN;
```

8.5 OTHER OUTCOME MEASURES

8.5.1 TIME SPAN UNTIL PHYSICIAN CONTACT AND HOSPITAL ARRIVAL

The outcome measures time span until first physician contact and time span until arrival at hospital are defined as a time variables that are calculated by:

```
/* Other outcome measure: time span until first physician contact */
DATA temsData; set temsData;
        if _1010_group=2 then do;
                if _502_proto=1 then do;
                        if _1002_konv=1 then do;
                                time_physician=mod((_6012_TNA -
                                _6601_time_alarm1 + '24:0't),'24:0't); end;
                        else if _1002_konv ne 1 then do;
                                time_physician=mod((_6602_time_arrival_scene -
                                _6601_time_alarm1 + '24:0't),'24:0't); end;
                        else do; time_physician=.; end; end;
                else if _502_proto=2 then do;
                        if _503_proto=1 then do;
                                time_physician=mod((_6009_RTW -
                                _6601_time_alarm1 + '24:0't),'24:0't); end;
                        else do; time_physician=.; end; end;
                else do; time_physician=.; end; end;
        else if _1010_group=1 then do;
                if _502_proto=1 then do;
                        if _1002_konv=1 then do;
                                time_physician= mod((min(_6012_TNA-_6011_TNA1,
                                _6602_time_arrival_scene-_6011_TNA1) + '24:0't),'24:0't); end;
                        else if _1002_konv ne 1 then do;
                                time_physician=mod((_6012_TNA -
                                _6011_TNA1 + '24:0't),'24:0't); end;
                        else do; time_physician=.; end; end;
                else if _502_proto=2 then do;
                        if _503_proto=1 then do;
                                time_physician=mod((_6009_RTW -
                                _6011_TNA1 + '24:0't),'24:0't); end;
                        else do; time_physician=.; end; end;
                else do; time_physician=.; end; end;
        else do; time_physician=.; end;
        format time_physician hhmm4.;
RUN;
/* Other outcome measure: time span until arrival at hospital */
DATA temsData; set temsData;
        if _1120_transport=1 then do;
                time_arrival=_6009_RTW-_6006_RTW1; end;
        else do time_arrival=.; end;
RUN;
```

8.5.2 CONVERSION OF EMS PHYSICIAN TO TELE-EMS PHYSICIAN

The outcome measure conversion of EMS-physician to tele-EMS-physician is a status variable that is calculated by:

```
/*Conversion of EMS physician to tele-EMS physician*/
DATA temsData; set temsData;
        if _1010_group=2 and _1002_konv then konv_EMS=1;
        else konv_EMS=0;
RUN;
```

8.6 UNBLINDING

There are no events expected to require unblinding of the outcome assessors.

8.7 DERANDOMISATION

Not applicable.

9. DESCRIPTIVE STATISTICS

All results will be rounded to one decimal place more than decimal places are given in Case Report Form. Percentages will be rounded to one position after decimal point.

Because of possible rounding errors the sum of percentages is not necessary 100 %.

9.1 BASELINE CHARACTERISTICS & OTHER OUTCOME MEASURES

Descriptive statistics of all continuous baseline characteristics include the number of available observations (N), mean and standard deviation (SD) separated by treatment group. Descriptive statistics of all dichotomous or categorical baseline characteristics include the number of available observations (N), frequency (n) and percentage (%) separated by treatment group. A possible tabular summary of baseline characteristics is shown in Table 5.

Characteristic	Conventional EMS Physician		Tele-EMS physician	
	N	Statistical measure	N	Statistical measure
Sex (female)				
Time span until first physician contact				
Time span until arrival at hospital				
NACA classification first diagnosis (in case of physician contact)				
Not documented				
NACA 2				
NACA 3				
NACA 4				
NACA 5				
NACA 6				
NACA 7				
NACA classification last diagnosis (in case of physician contact)				
Not documented				
NACA 2				
NACA 3				
NACA 4				
NACA 5				
NACA 6				
NACA 7				

Table 5: Descriptive statistics of different types of baseline characteristics

9.2 VARIABLES EVALUATED FOR EFFICACY

9.2.1 PRIMARY ENDPOINT

Descriptive analysis of the dichotomous primary endpoint and its separate components includes description of number of available observations (N), frequency (n) and percentage (%) separated by treatment group like described in Table 6.

Variable	Conventional EMS Physician		Tele-EMS physician	
	N	n (%)	N	n (%)
Immediate allergic reaction to drug application due to incorrect survey of patients` medical history (omission of the question for drug allergies)				

Intervention-related and immediate treatment-requiring blood pressure drop on scene (e.g. after wrong drug dosage or drug selection).	
Immediate intervention-related apnea or respiratory insufficiency on scene (e.g. after wrong drug dosage or drug selection).	
Intervention-related circulatory arrest within 24h of EMS treatment (e.g. after wrong drug dosage, drug selection, or wrong hospital referral).	

Table 6: Descriptive statistics of dichotomous primary endpoint components

The presentation of further statistics of the primary endpoint is described in section 10.1.

SECONDARY ENDPOINTS

9.2.1.1 DICHOTOMOUS AND CATEGORICAL VARIABLES

Descriptive statistics of dichotomous secondary endpoints includes description of number of available observations (N), frequency (n) and percentage (%) separated by treatment group like described in Table 7.

Variable	Conventional EMS Physician		Tele-EMS physician	
	N	n (%)	N	n (%)
Quality of medical history survey (adherence to SAMPLER)				
S - Symptoms				
A - Allergies				
M - Medication				
P - Past medical history				
L - Last oral intake				
E - Events prior to incident				
R - Risk factors				
Treatment quality (adherence to guidelines)				
Correct hospital choice				
ECG used				
NIBP measured				
SpO2 measured				
Glucose measured				
IV cannula placed				
Frequency of predefined "Tracer" diagnoses, with physician contact (yes)				
Trauma				
Stroke				
Acute coronary syndrome				
Pain control				
Bronchial asthma				
Chronic obstructive pulmonary disease (COPD)				
Seizure				
Sepsis				
Hypoglycemia				

At least one AE per patient (independently of the kind of EMS care, e.g. allergic reaction despite adequate survey of medical history, not-intervention related blood pressure dropping, apnea or cardiac arrest or other)	
Other intervention-related AEs	
Premature termination of the telemedical or conventional EMS operation, as unnecessary (yes)	
Required conversion from the primary dispatched tele-EMS physician to a conventional EMS physician (yes)	
Assessment if a conventional EMS physician operation could have been handled by a tele-EMS physician (yes)	
Death within 24 h	
Death within 30 days	
Death within 90 days	
Death until hospital discharge	

Table 7: Descriptive statistics of dichotomous secondary endpoints

9.2.1.2 CONTINUOUS AND QUASI CONTINUOUS VARIABLES

Descriptive statistics of continuous and quasi continuous secondary endpoints includes description of number of available observations (N), mean and standard deviation (SD) or median, as well as lower quartile (Q1) and upper quartile (Q3) in the case of heavily skewed data like described in Table 8. The skewness of continuous endpoints is investigated using histograms and boxplots.

Variable	Conventional EMS Physician		Tele-EMS physician	
	N	Statistical Measure	N	Statistical Measure
Quality of medical history survey (adherence to SAMPLE)				
Quality of documentation				
Duration of the physician engagement-time				
Hospital length of stay				
Intensive care unit length of stay				

Table 8: Descriptive statistics of continuous and quasi continuous secondary endpoints

9.3 VARIABLES EVALUATED FOR SAFETY

Compare primary and secondary endpoints.

9.4 VARIABLES EVALUATED FOR COMPLIANCE

Not applicable.

9.5 GRAPHICAL PRESENTATION

All separate components of primary endpoint (AE classes) as well as binary secondary endpoints will be presented by two-sided 95%-confidence interval for the odds ratio of separate secondary endpoints of both treatment groups.

Continuous outcomes as time intervals will be presented by boxplots.

10. INFERENTIAL STATISTICS

10.1 PRIMARY ENDPOINT

DEFINITION

The primary outcome measure will include the rate of AEs depending on the specific treatment by an EMS physician or a tele-EMS physician. A Clinical Endpoint Committee (CEC) will perform the endpoint adjudication by blinded evaluation and assignment of AEs into intervention-related AEs, according to predefined criteria.

These intervention-related AEs are defined as follows:

- Immediate allergic reaction to drug administration due to incorrect survey of patients' medical history (omission of the question for drug allergies).
- Intervention-related and immediate treatment-requiring hypotensive episode on scene (e.g., after wrong drug dosing or drug selection).
- Immediate intervention-related apnea or respiratory insufficiency on scene (e.g., after wrong drug dosing or drug selection).
- Intervention-related circulatory arrest within 24 h of EMS treatment (e.g., after wrong drug dosing, drug selection, or wrong referral to a hospital, which is not-specialized for the respective emergency case).

HYPOTHESES

The evaluation of the primary endpoint "rate of intervention related AEs" implies testing the hypothesis:

$$H_0: p_{EMS-physician} - p_{tele-EMS} \leq -0.015$$

The rate of intervention related AEs in the tele-EMS(experimental)group is inferior to the EMS-physician (control)-group by more than $\delta = 0.015$.

versus

$$H_1: p_{EMS-physician} - p_{tele-EMS} > -0.015.$$

The rate of intervention related AEs in the tele-EMS(experimental)group is <u>not</u> inferior to the EMS-physician (control)-group by more than $\delta = 0.015$.

STATISTICAL EVALUATION

The null hypothesis of the primary endpoint $H_0: p_2 - p_1 \leq -0.015$ (non-inferiority) will be tested by the confidence interval of rate differences. Because in general neither the ITT nor the PP population will give a conservative test decision for a non-inferiority study, the hybrid-population after Sanchez (Sanchez 2006) will be used. Therefor the limits of the significance level will be chosen as described in Table 10.

```
/*Analysis of the primary endpoint*/
PROC FREQ data=temsData;
      tables _1010_group*ae /riskdiff alpha=0.0225 CL;
RUN;
```

10.2 SECONDARY ENDPOINTS

10.2.1 DICHOTOMOUS SECONDARY ENDPOINTS

DEFINITION

The following dichotomous secondary endpoints will be evaluated by inferential statistics:

- Non-intervention related adverse event
- Death within 24 hours
- Death within 30 days
- Death within 90 days
- Death until hospital discharge

HYPOTHESES

$$H_0: p_{EMS-physician} = p_{tele-EMS}$$

The rates of the special secondary endpoint are equal under both interventions.

versus

$$H_1: p_{EMS-physicia} \neq p_{tele-EMS}$$

The rates of the special secondary endpoint are different under both interventions.

STATISTICAL EVALUATION

For each of the dichotomous secondary endpoints a two-sided chi-square test will be conducted.

```
/*Analysis of dichotomous secondary endpoints*/
PROC FREQ data=temsData;
	tables _1010_group*dich_sec_ep /chisq;
RUN;
```

10.2.2 CONTINUOUS AND QUASI CONTINUOUS SECONDARY ENDPOINTS

DEFINITION

The following continuous or quasi continuous secondary endpoints will be evaluated by inferential statistics:

- Quality of medical history survey (SAMPLE)
- Quality of documentation
- Physician engagement time
- Hospital length of stay
- Intensive care unit length of stay

HYPOTHESES

$$H_0: \mu_{EMS-physician} = \mu_{tele-EMS}$$

The means of the special secondary endpoint are equal under both interventions.

versus

$$H_1: \mu_{EMS-physician} \neq \mu_{tele-EMS}$$

The means of the special secondary endpoint are different under both interventions.

STATISTICAL EVALUATION

For each of the continuous or quasi continuous secondary endpoints a two-sided t-test will be conducted.

```
/*Analysis of continuous or quasi continuous secondary endpoints*/
PROC TTEST data=temsData;
       class _1010_group;
       var cont_sec_ep;
RUN;
```

10.3 MULTIPLICITY

There is only one primary endpoint, no α-adjustment is necessary.

All secondary endpoints are evaluated exploratively, whereby a 5% significance level is assumed.

10.4 SAMPLE SIZE AND STATISTICL POWER

The sample size calculation is based on an assumed AE rate of 2% for the conventional physician-based EMS. The rate of 2% is based on our own analysis of 100 EMS-physician cases, as we could not find any information about EMS-related AEs in the medical literature. We assumed a non-inferiority margin of 1.5% and allocated the overall 5% significance level to K = 3 (power (1 – β) 80.0%). Interim analysis will be performed according to the procedure of O'Brien und Fleming (O'Brian 1979). The critical values, power and sample sizes for the group sequential design are given in Table 9, using Addplan 6.0 (http://www.aptivsolutions.com/addplan-software/).

Using an allocation ratio of $(n_2/n_1) = 1$, the necessary sample size is 1504.2 + 1504.2 = 3008.4, thus resulting in a total sample size of 3010 patients. A fixed sample size design would need n_1 = 1478.5 and n_2 = 1478.5. The expected total sample size under the alternative is 2531.7. A stop for futility is not planned.

Information rate	Bounds accept H_0	Bounds reject H_0	Significance level one-sided	α spent	β spent	Power achieved	Stage n_1	Stage n_2
0.333	-	3.471	0.0003	0.0003	-	0.0329	501.4	501.4
0.667	-	2.454	0.0071	0.0072	-	0.4424	501.4	501.4
1.0	2.004	2.004	0.0225	0.025	-	0.8000	501.4	501.4

Table 9: Statistical calculation based on Farrington und Manning formula (Farrington 1990). H0=null hypothesis, n=number.

10.5 INTERIM ANALYSES

Two interim analyses will be performed after 33%, 67% and 100% of the patients are evaluable. Here the primary endpoint will be evaluated by the Safety Committee for discussion of possible consequences for the continuation of the trial. Interim analysis will be performed according to the O'Brien Fleming procedure (O'Brian 1979).

At the interim analyses the statistical test will use the significance level described in Table 10.

Stage	Information rate	Significance level (one-sided)
1	0.333	0.0003
2	0.667	0.0071
3	1.0	0.0225

Table 10: Significance levels of the O'Brian Flemming (O'Brian 1979) Interim Analysis (K=3)

10.5.1 DECISION RULES OF INTERIM ANALYSES

If there appears to be a difference of 5% intervention-related AEs in one study-arm, then the DSMC must recommend premature study termination.

11. FUTURE ANALYSES IN EXTENSION TO THE ACTUAL EVALUATION

In addition to the actual evaluation of the TEMS data described here, future work will be carried out on the evaluation of the following subgroup analyses, for example in the context of doctoral theses:

- Accordance of the prehospital diagnosis with the discharge diagnosis
- Quality of care: overall quality of anamnesis, diagnostics and treatment
- Quality of care: specific quality of, diagnostics and treatment of stroke
- Quality of care: specific quality of diagnostics and treatment of acute coronary syndrome
- Quality of care: specific quality of diagnostics and treatment of cardiac arrhythmia
- Quality of care: specific quality of diagnostics and treatment of hypertensive emergency
- Quality of care: specific quality of diagnostics and treatment of pain
- Quality of care: specific quality of diagnostics and treatment of hypoglycemia
- Outcome quality: Hospital and ICU length of stay, hospital/30 days/90 days survival
- Technical performance of the tele-EMS system
- Economic aspects: physician engagement time
- Acceptance of the teleconsultation system by the involved EMS and emergency room physicians
- Acceptance of the teleconsultation system by the paramedics
- Acceptance of the teleconsultation system by the patients
- Retrospective analysis of the same data (excluding the prospectively collected satisfaction surveys and 30- and 90-day follow-up), as in the main study for the excluded conventional EMS physician cases.
- Additionally, all outcomes will be assessed for patients who recruited during the run-in phase

12. PREPARATION AND QUALITY CONTROL OF PROGRAM CODE

12.1 STORAGE OF LOG FILES

All programs are stored in V:\KlinischeStudien\TEMS\Auswertung\Programme.

SAS-Log and SAS-Output will be controlled and stored in

V:\KlinischeStudien\TEMS\Auswertung\Ergebnisse.

12.2 CONTROL OF PROGRAM

SAS-MACROS and SAS programs for data transformation will be controlled by test data set.
Four-eyes principle will be applied to control SAS-Code.
In all SAS programs comments will be added.

13. CHANGES FROM STUDY PROTOCOL

No changes are defined.

14. REFERENCES

Farrington, CP, and Manning, G. „Test statistics and sample size formulae for comparative binomial trials with null hypothesis of non-zero risk difference or non-unity relative risk." *Statistics in Medicine. 9.*, 1990: 1447-1454.

O'Brian, PC, and Flemming, TR. „A Multiple Testing Procedure for Clinical Trials." *Biometrics. 35.*, 1979: 549-556.

Sanchez, M, and Chen, X. „Choosing the analysis population in non-inferiority studies: per protocol or intention-to-treat." *Statistics in medicine.25.*, April 2006: 1169-81.

15. SIGNATURES

Agreement to the Trial Statistical Analysis Plan:

Principal Investigator (gem §4 Abs. 25 AMG):

[signature]	Aachen, 9.7.2020
Univ. Prof. Dr. med. Rolf Rossaint	Place / Date

Biostatistician:

[signature]	AACHEN, 8.7. 2020
Univ. Prof. Dr. rer. nat. Ralf-Dieter Hilgers	Place / Date
