# Supplementary material for: Telemedical support for prehospital emergency medical service in severe emergencies: an open-label randomised non-inferiority clinical trial
Source: Crit Care. 2023 Jun 30;27:256. doi: 10.1186/s13054-023-04545-z (PMC10311733; doi:10.1186/s13054-023-04545-z)
Supplement: Supplementary file 3 — Additional file 3. eMethods. eResults. eTable 1. Other Baseline characteristics. eTable 2. Frequency of predefined “Special” diagnoses, with physician contact. eTable 3. Frequency of main diagnoses with physician contact. eTable 4. Other secondary outcomes. [file 13054_2023_4545_MOESM3_ESM.docx]

Supplementary materials to

**Telemedical support for prehospital emergency medical service in severe emergencies: An open-label randomised non-inferiority clinical trial**

Ana Kowark,^1,2^ * Marc Felzen,^1^ * Sebastian Ziemann,^1^ Stephanie Wied,^3^ Michael Czaplik,^1^ Stefan K. Beckers,^1^ Jörg Christian Brokmann,^4^ Ralf-Dieter Hilgers, ^3§^ Rolf Rossaint,^1 # §^ on behalf of the TEMS-study group

*contributed equally as first authors

^§^contributed equally as last authors

^#^corresponding author

**Corresponding Author**

Rolf Rossaint, MD, Department of Anaesthesiology, Medical Faculty University Hospital RWTH Aachen, Pauwelsstr. 30, 52074 Aachen, Germany

Email: rrossaint@ukaachen.de, Telephone: +49-241-8088179.

**Supplement 3.**

**Content Supplementary Online Content 3**

**eMethods.**

**eResults.**

**eTable 1.** Other Baseline characteristics

**eTable 2.** Frequency of predefined “Special” diagnoses, with physician contact

**eTable 3.** Frequency of main diagnoses with physician contact

**eTable 4.** Other secondary outcomes

This supplementary material has been provided by the authors to give readers additional information about their work.

**Table of content**

Supplementary Methods 4

Ethics statement 4

Teleconsultation system 4

Randomisation, study procedure, and interventions 4

Tele and control groups informed consent for Follow-up 4

Outcomes 4

Data quality 4

Blinding 4

Statistical analysis 5

Interim analyses 5

Evaluation of the primary endpoint 5

Supplementary Results 7

Patient characteristics 7

eTable 1. Other Baseline characteristics. 8

eTable 2. Frequency of predefined “Special” diagnoses, with physician contact 11

eTable 3. Frequency of main diagnoses with physician contact 12

eTable 4. Other secondary outcome 13

# Supplementary Methods

## Ethics statement

The institutional ethics committee of the University Hospital Aachen has approved (EK 170/15) this trial on 25 November 2015. The ethics committee waived an informed consent for the first step of the trial, the randomisation and assignment to the treatment group, as it occurs before any patient contact to a physician and thus was not feasible. Written informed consent was required and provided at least at hospital admission by all patients who were followed up after randomisation and initial treatment with one of the two EMS.

## Participants

The exclusion criteria comprised according to the study protocol:^19^ All life-threatening emergency cases, where a physically present EMS physician on scene is obligatory required according to German regulations. These include patient condition and emergency case related indications:

a) Patient condition related indications:

• Apnoea

• Acute respiratory failure

• Cardio-circulatory arrest

• ST-elevation myocardial infarction (STEMI)

• Unconsciousness

• Persistent seizure

• Life- threatening rhythm disorder

• Major trauma

• Complex psychiatric disorders

• Age < 18 years

b) Emergency case related indications

• Major vehicle accident

• (Traffic) accident with children

• Fall from a height (> 3m)

• Gunshot-, stab-, or blow injuries in the head, neck and torso area

• Fires with reference to personal injury

• Explosion-, thermic or chemical accidents with reference to personal severe injury

• High-voltage electrical accident

• Water connected accidents (drowning-, diving accident, fall through ice)

• Accidents involving hazardous goods

• Hostage-taking, rampage or other crimes with potential danger for human life (preventive deployment, police consultation)

• Immediate threatening suicide

• Immediate forthcoming delivery or preceding delivery

## Teleconsultation system

The teleconsultation system consists of a mobile audio connection and transmission of patients’ real-time vital data (e.g., 12-lead electrocardiography, pulse oxymetry, and non-invasive blood pressure) from the emergency scene to an experienced tele-EMS physician located in a control centre. Further, a unidirectional video stream and photo transmission are enabled from the ambulance cabin to the tele-EMS physician. The tele-EMS physician in the control centre uses specifically developed software; after making a working diagnosis, the physician is prompted with a guideline-based standard operating procedure, including a corresponding treatment algorithm and corresponding checklist.

## Randomisation, study procedure, and interventions

The distinction between severe and life-threatening cases was automatically made by the dispatching software according to an implemented algorithm.

## Tele and control groups informed consent for Follow-up

Patients who were not admitted to the hospital and remained at the scene could not be followed up to avoid performance bias, as the remote physicians could not personally seek the written informed consent for follow-up procedures.

## Outcomes

The term intervention-related covered beside wrong treatments also the cases where the respective EMS did not provide any treatment. The predefined AEs comprised immediate new iatrogenic allergic reaction (new allergic reaction during the EMS mission documented in the free text or indicated by the use of anti-allergic drugs like e.g. antihistaminic drugs or corticoids), new iatrogenic decrease in blood pressure (necessity of vasopressors), new iatrogenic respiratory insufficiency (decrease in peripheral oxygen saturation <90%, or need for assistive, manual or controlled ventilation), and cardiac arrest within 24 hours of the intervention. Iatrogenic meant that the treating team induced the complications.

In order to ensure blinding of the Clinical Endpoint Committee (CEC) all EMS files were transcribed into a neutral excel database without disclosure of the group assignment. The CEC consisted of fully independent persons without any link to the study team.

The outcome assessors received an extensive training and guidelines.

## Data quality

The principal investigators in the participating hospitals ensured adherence to the in-hospital study protocol as well as the accuracy of data entries in the database. Qualified on-site monitoring was performed.

## Blinding

Only the assessors of the 30- and 90-day follow-ups were blinded to the treatment group. They contacted the patients according to a patient list on an Excel sheet (Microsoft Inc.) that did not include the allocated intervention. They were also instructed not to ask patients which group they were in. The assessors only assessed the survival status and the Modified Rankin Scale score (at 90 days follow-up), which assesses disability in patients who have had a stroke.

## Statistical analysis

### Interim analyses

Because of organisational difficulties in deviation from the study protocol and trial statistical analysis plan (TSAP) (Supplement 2), the judgment of the Clinical Endpoint Committee (CEC) regarding the causality of the adverse events (AEs) to the group assignment could not be performed before the interim analyses; thus, all AE cases were included in the interim analyses of the primary endpoint.

The first and second interim analyses were conducted after 1115 and 2229 patients, respectively, were enrolled and recorded in the database. The null hypothesis of non-inferiority cannot be rejected in either case.

Telemedical support for pre-hospital Emergency Medical Service (TEMS) Trial – First Interim Analysis

Overview:

|  | Primary Endpoint: Adverse event occurred during emergency operation | | Both groups (n) |
| --- | --- | --- | --- |
|  | Control group (n) | Tele group (n) |  |
| No | 591 | 454 | 1045 |
| Yes | 7 | 6 | 13 |
| Total | 598 | 460 | 1058 |

- n=1115 recorded emergency cases

- n=1058 emergency cases with available data on the primary endpoint (n=34 missing emergency cases for the primary endpoint in the control group, n=23 emergency cases missing for the primary endpoint in the tele group)

Statistical Evaluation:

The null hypothesis 𝐻0: 𝑝𝐸𝑀𝑆−𝑝h𝑦𝑠𝑖𝑐𝑖𝑎𝑛−𝑝𝑡𝑒𝑙𝑒−𝐸𝑀𝑆 ≤−0.015 (non-inferiority) for the primary endpoint was tested by the asymptotic confidence interval of rate differences. A one-sided significance level of 0.0003 was used according to the O’Brien Fleming procedure.^1^

The 99.97% confidence interval for the rate difference 𝑝𝐸𝑀𝑆−𝑝h𝑦𝑠𝑖𝑐𝑖𝑎𝑛−𝑝𝑡𝑒𝑙𝑒−𝐸𝑀𝑆 was calculated as -0.0262, 0.0235. Therefore, the null hypothesis of non-inferiority cannot be rejected at a significance level of 0.0003.

TEMS Trial – Second Interim Analysis

Overview:

|  | Primary Endpoint: Adverse event occurred during emergency operation | | Both groups (n) |
| --- | --- | --- | --- |
|  | Control group (n) | Tele group (n) |  |
| No | 1166 | 959 | 2125 |
| Yes | 11 | 12 | 23 |
| Total | 1177 | 971 | 2148 |

- n=2229 recorded emergency cases

- n=2148 emergency cases with available data on the primary endpoint (n=37 missing emergency cases for the primary endpoint in the control group, n=44 missing emergency cases for the primary endpoint in the tele group)

Statistical Evaluation:

The null hypothesis 𝐻0: 𝑝𝐸𝑀𝑆−𝑝h𝑦𝑠𝑖𝑐𝑖𝑎𝑛−𝑝𝑡𝑒𝑙𝑒−𝐸𝑀𝑆 ≤−0.015 (non-inferiority) for the primary endpoint was tested by the asymptotic confidence interval of rate differences. A one-sided significance level of 0.0071 was used according to the O’Brien Fleming procedure.^1^

The 99.29% confidence interval for the rate difference 𝑝𝐸𝑀𝑆−𝑝h𝑦𝑠𝑖𝑐𝑖𝑎𝑛−𝑝𝑡𝑒𝑙𝑒−𝐸𝑀𝑆 was calculated as -0.0152, 0.0092. Therefore, the null hypothesis of non-inferiority cannot be rejected at a significance level of 0.0071.

### Evaluation of the primary endpoint

The causality of the primary endpoint (intervention-related AE) to the group assignment was planned from the beginning of the study to be dichotomously judged by the CEC by the attributes “suspected” or “not suspected”, as a proof of causality was not possible and it was not a judgment about the relationship of an AE and an investigational product. However, the variable “CEC decision” was erroneously coded in the OpenClinica database with five attributes and the description of the primary endpoint in the first TSAP version based on this coding. Following the first original version of the TSAP, an AE had only a reasonable causal relationship if “certain” was ticked in OpenClinica. “Suspected” was not an available attribute. Therefore, we had to amend the TSAP as follows: an AE was “suspected” to have a causal relationship if either the attribute “certain” or “probable” were ticked in the database. Other minor changes in TSAP levels are presented in Supplement 2.

**Reference**

1. O'Brien PC, Fleming TR. A multiple testing procedure for clinical trials. *Biometrics*. 1979;35(3):549-556.

# Supplementary Results

## Patient characteristics

Most patients had an initial patient severity score adapted from the Eight-step National Advisory Committee for Aeronautics of 3 (severe but not life-threatening). The most frequent initially suspected diagnosis entered into the dispatching software by the dispatching personnel was acute coronary syndrome (388/3220, 12.1%).

# eTable 1. Other Baseline characteristics.

|  | **All (n = 3220)** | **Control group (n = 1676)** | **Tele-group (n = 1544)** |
| --- | --- | --- | --- |
| Discharge destination, No. (%)^a^ |  |  |  |
| home | 2344 (72.8) | 1226 (73.2) | 1118 (72.4) |
| other hospital | 127 (3.9) | 68 (4.1) | 59 (3.8) |
| rehabilitation facility | 44 (1.4) | 20 (1.2) | 24 (1.6) |
| death | 59 (1.8) | 25 (1.5) | 34 (2.2) |
| other | 64 (2.0) | 29 (1.7) | 35 (2.3) |
| missing data | 582 (18.1) | 308 (18.4) | 274 (17.6) |
| Proportion of conversions from the assigned to the other group, No. (%) | 122 (3.8) | 20 (1.2)^b^ | 99 (6.4)^c^ |
| Medical education of the treating physicians |  |  |  |
| Since approval of medical license, mean (SD), years | 11.6 (5.7) | 13.6 (6.7) | 10.0 (4.1) |
| Since approval as EMS physician, mean (SD), years | 8.1 (6.3) | 10.5 (8.0) | 6.0 (3.6) |
| Necessity of a Tele-EMS physician for the respective emergency case, No. (%) |  | NA |  |
| Missing data | 1823 (56.5) | 1672 (99.8) | 151 (9.8) |
| No, only paramedics would have been needed in view of the Tele-EMS physician | 93 (2.9) | 0 (0) | 93 (6.0) |
| No, not needed in view of the paramedics and thus the Tele-EMS physician was not contacted | 810 (25.2) | 1 (0.1) | 809 (52.4) |
| Yes, drug application and thus the prescription of drugs was necessary | 246 (7.6) | 2 (0.1) | 244 (15.8) |
| Yes, the knowledge of the Tele-EMS physician was necessary | 175 (5.4) | 1 (0.1) | 174 (11.3) |
| Yes, for bridging until the arrival of a conventional EMS physician | 18 (0.6) | 0 (0) | 18 (1.2) |
| Yes, for support of the conventional EMS physician | 2 (0.1) | 0 (0) | 2 (0.1) |
| Yes, for other reason | 53 (1.7) | 0 (0) | 53 (3.4) |
| Necessity of a conventional EMS physician for the respective emergency case, No. (%) | NA |  |  |
| Missing data | 2124 (66.0) | 619 (36.9) | 1504 (97.5) |
| No, only paramedics would have been needed in view of the conventional EMS physician | 443 (13.5) | 431 (25.7) | 2 (0.1) |
| No, a tele-EMS physician would have been enough in view of the conventional EMS physician | 477 (14.8) | 465 (27.7) | 12 (0.8) |
| Yes, intravenous access was difficult | 45 (1.4) | 36 (2.2) | 9 (0.6) |
| Yes, patient was unstable and needed a physician on scene | 55 (1.7) | 49 (2.9) | 6 (0.4) |
| Yes, manual skills on scene were necessary^d^ | 6 (0.2) | 5 (0.3) | 1 (0.1) |
| Yes, specific psychiatric evaluation necessary (restricted by law) | 1 (0.1) | 1 (0.1) | 0 (0) |
| Yes, for other reason | 61 (1.9) | 53 (3.2) | 8 (0.5) |
| No, a conventional EMS-physician was not necessary in view of the paramedics | 18 (0.6) | 17 (1.0) | 1 (0.1) |
|  |  |  |  |
| NACA score first diagnosis^a, e^, No. (%) | n = 1867^f^ | n = 1308^f^ | n = 559^f^ |
| NACA 2 - moderate disturbance, no hospital admission necessary | 266 (12.0) | 200 (12.8) | 66 (10.1) |
| NACA 3 - severe but not life-threatening | 1192 (53.7) | 837 (53.4) | 355 (54.5) |
| NACA 4 - potentially life-threatening | 345 (15.6) | 237 (15.1) | 108 (16.6) |
| NACA 5 - acute risk of death | 57 (2.6) | 31 (2.0) | 26 (4.0) |
| NACA 6 - successful cardiopulmonary resuscitation | 4 (0.2) | 3 (0.2) | 1 (0.2) |
| NACA 7 - death | 3 (0.1) | 0 (0) | 3 (0.5) |
| NACA score last diagnosis, No. (%)^a, e^ | n = 1763 | n = 1217 | n = 546 |
| NACA 2 - moderate disturbance, no hospital admission necessary | 264 (11.9) | 198 (12.6) | 66 (10.1) |
| NACA 3 - severe but not life-threatening | 1189 (53.6) | 835 (53.3) | 354 (54.4) |
| NACA 4 - potentially life-threatening | 252 (11.4) | 158 (10.1) | 94 (14.4) |
| NACA 5 - acute risk of death | 49 (2.2) | 21 (1.3) | 28 (4.3) |
| NACA 6 - successful cardiopulmonary resuscitation | 3 (0.1) | 1 (0.1) | 2 (0.3) |
| NACA 7 - death | 6 (0.3) | 4 (0.3) | 2 (0.3) |

Abbreviations=AEs, adverse events; CI=confidence interval; EMS=emergency medical service; NA=not applicable; NACA=National Advisory Committee for Aeronautics; No.=number; SD=standard deviation

^a^ Percentages may not add to 100% due to rounding.

^b^ Emergency cases assigned to the control group, which were passed to a tele-EMS physician either by the conventional EMS physician or manually by the personnel in the dispatching centre.

^c^ Emergency cases assigned to the tele-group, which were passed to a conventional EMS physician either by the tele-EMS physician or manually by the personnel in the dispatching centre.

^d^ For example: intubation.

^e^ Only applicable for the cases with physician contact, as it is not assessed in the paramedic EMS files.

^f^ Total number is deviating from the total number indicated in the column heading due to missing data.

# eTable 2. Frequency of predefined “Special” diagnoses, with physician contact

|  | **Control group (n = 1568)** | **Tele-group (n = 651)** | **Risk difference (95% CI)** | ***P* value** |
| --- | --- | --- | --- | --- |
| Trauma | 90 (5.7) | 29 (4.5) | -1.3 (-3.2–0.7) | 0.221 |
| Stroke | 143 (9.1) | 93 (14.3) | 5.2 (2.1–8.2) | <0.001 |
| Acute coronary syndrome | 188 (12.0) | 115 (17.7) | 5.7 (2.3–9.0) | <0.001 |
| Pain control | 466 (29.7) | 180 (27.7) | -2.1 (-6.2–2.0) | 0.329 |
| Bronchial asthma | 8 (0.5) | 4 (0.6) | 0.1 (-0.6–0.8) | 0.755 |
| Chronic obstructive pulmonary disease | 54 (3.4) | 21(3.2) | -0.2 (-1.9–1.4) | 0.796 |
| Seizure | 42 (2.7) | 23 (3.5) | 0.9 (-0.8–2.5) | 0.277 |
| Sepsis | 160 (10.2) | 68 (10.5) | 0.2 (-2.6–3.0) | 0.865 |
| Hypoglycaemia | 24 (1.5) | 5 (0.8) | -0.8 (-1.7–0.1) | 0.004 |

Specific diagnoses, which were chosen by the authors as important diagnoses within the emergency medical service.

# eTable 3. Frequency of main diagnoses with physician contact

|  | **Conventional EMS physician (n = 1568)** | **Tele-EMS physician (n = 651)** | **Risk difference (95% CI)** | ***P* value** |
| --- | --- | --- | --- | --- |
| **Frequency of main diagnoses, No. (%)^a^** | **Control group** | **Treatment group** |  |  |
| Cardiovascular disorder^b^ | 297 (18.9) | 104 (16.0) | -3.0 (-6.4–0.5) | 0.098 |
| Abdominal disorder^c^ | 240 (15.3) | 52 (8.0) | -7.3 (-10.1– -4.6) | <0.001 |
| Neurological disorder^d^ | 208 (13.3) | 126 (19.4) | 6.1 (2.6–9.6) | <0.001 |
| Acute coronary syndrome | 172 (11.0) | 99 (15.2) | 4.2 (1.1–7.4) | 0.006 |
| Orthopaedic | 112 (7.1) | 54 (8.3) | 1.2 (-1.3–3.6) | 0.348 |
| Pulmonary disorder^e^ | 108 (6.9) | 48 (7.4) | 0.5 (-1.9–2.9) | 0.684 |
| Rhythm disorder | 81 (5.2) | 53 (8.1) | 3.0 (0.6–5.3) | 0.007 |
| Trauma | 69 (4.4) | 25 (3.8) | -0.6 (-0.2–1.2) | 0.551 |
| Acute abdomen^f^ | 68 (4.3) | 19 (2.9) | -1.4 (-3.1–0.2) | 0.117 |
| Intoxication | 42 (2.7) | 11 (1.7) | -1.0 (-2.3–0.3) | 0.165 |
| Psychiatric disorder | 40 (2.6) | 12 (1.8) | -0.7 (-2.0–0.6) | 0.316 |
| Others | 29 (1.8) | 15 (2.3) | 0.5 (-0.9–1.8) | 0.484 |
| Metabolic disorder | 22 (1.4) | 9 (1.4) | -0.0 (-1.1–1.1) | 0.970 |
| Anaphylaxis | 9 (1.4) | 17 (1.1) | 0.3 (-0.7–1.3) | 0.552 |
| Reduced general condition | 11 (0.7) | 2 (0.3) | -0.4 (-1.0–0.2) | 0.368 |
| Missing data | 60 (3.8) | 5 (0.8) | NA | NA |

NA=not applicable; No.=number.

^a^ Percentages may not add to 100% due to rounding.

^b^ The category “Cardiovascular disorder” comprises among others: arterial hypertension, syncope, dehydration, pulmonary embolism, cardiopulmonary arrest.

^c^ The category “Abdominal disorder” comprises among others: gastroenterological disorders, urological disorders and gynaecological disorders.

^d^ The category “Neurological disorder” comprises among others: strokes and seizures.

^e^ The category “Pulmonary disorder” comprises among others: chronic obstructive pulmonary disease, bronchial asthma and pneumonia.

^f^ The category “Acute abdomen” comprises among others: ileus and acute diverticulitis.

# eTable 4. Other secondary outcome

|  | **Control group (n = 1676)** | **Tele-group**  **(n = 1544)** | **Mean difference or Risk difference^f^ (95% CI)** | ***P* value** |
| --- | --- | --- | --- | --- |
| At least one immediate non-intervention related AE per patient, No. (%)^a, b^ | 14 (0.8) | 15 (1.0) | 0.1 (-0.5–0.8) | 0.683 |
| Non-intervention-related allergic event, No. (%) ^a, b^ | 1 (0.1) | 0 (0) | -0.1 (-0.2–0.1) | >0.99 |
| Non-intervention related blood pressure drop, No. (%) ^a, b^ | 4 (0.2) | 3 (0.2) | 0.0 (-0.4–0.3) | >0.99 |
| Non-intervention related respiratory insufficiency, No. (%) ^a, b^ | 1 (0.1) | 1 (0.1) | 0.0 (-0.2–0.2) | >0.99 |
| Non-intervention related cardiac arrest within 24 hours of the intervention, No. (%) ^a, b^ | 9 (0.5) | 11 (0.7) | 0.2 (-0.4–0.7) | 0.527 |
| Non-intervention related other AE, No. (%) ^a, b^ | 0 (0) | 0 (0) | NA | NA |
| Other intervention-related AE, No. (%) ^a, b, c^ | 0 (0) | 0 (0) | NA | NA |
| Time span until first physician contact^e^ | n = 1507 ^d^ | n = 1188 ^d^ |  |  |
| mean (SD), min | 7.5 (5.4) | 27.3 (11.4) | 19.8 (19.1–20.5) | <0.001 |
| Time span until arrival at hospital^e^ | n = 1403 ^d^ | n = 1311 ^d^ |  |  |
| mean (SD), min | 37.4 (10.4) | 39.3 (13.9) | 1.8 (0.9–2.7) | <0.001 |

AE=adverse event; CI=confidence interval; EMS=emergency medical service; NA=not applicable; SD=standard deviation.

^a^ Percentages may not add to 100% due to rounding.

^b^ Non-intervention related means that the AE occurred independently of the EMS treatment, e.g. allergic reaction despite adequate survey of medical history, blood pressure decrease, apnoea or cardiac arrest or any other AE without connection to the treatment by the EMS personnel.

^c^ Other intervention-related AE means that the AE occurred iatrogenic, but it was not one of the four predefined AEs of the primary endpoint.

^d^ Total number is deviating from the total number indicated in the column heading due to missing data.

^e^ Time span is defined as the time between randomisation and the first physician contact, or arrival at hospital, respectively.

^f^ Mean difference for continuous variables and risk difference for categorical variables.
